# Developing a model to predict the early risk of hypertriglyceridemia based on inhibiting lipoprotein lipase (LPL): a translational study

**DOI:** 10.1038/s41598-023-49277-w

**Published:** 2023-12-19

**Authors:** Julia Hernandez-Baixauli, Gertruda Chomiciute, Juan María Alcaide-Hidalgo, Anna Crescenti, Laura Baselga-Escudero, Hector Palacios-Jordan, Elisabet Foguet-Romero, Anna Pedret, Rosa M. Valls, Rosa Solà, Miquel Mulero, Josep M. Del Bas

**Affiliations:** 1 Eurecat, Centre Tecnològic de Catalunya, Unitat de Nutrició i Salut, 43204 Reus, Spain; 2 Laboratory of Metabolism and Obesity, Vall d’Hebron-Institut de Recerca, Universitat Autònoma de Barcelona, Barcelona, Spain; 3 Eurecat, Centre Tecnològic de Catalunya, Centre for Omic Sciences (COS), Joint Unit Universitat Rovira i Virgili−EURECAT, 43204 Reus, Spain; 4 Functional Nutrition, Oxidation and Cardiovascular Diseases Group (NFOC-Salut), Facultat de Medicina i Ciències de la Salut, Universitat Rovira I Virgili, C/Sant Llorenç, 21, 43201 Reus, Spain; 5 Internal Medicine Service, Hospital Universitari Sant Joan de Reus, Av/del Doctor Josep Laporte, 2, 43204 Reus, Spain; 6 Nutrigenomics Research Group, Department of Biochemistry and Biotechnology, Universitat Rovira i Virgili, 43007 Tarragona, Spain; 7 Eurecat, Centre Tecnològic de Catalunya, Àrea Biotecnologia, Reus, Spain

**Keywords:** Biomarkers, Endocrine system and metabolic diseases, Cardiovascular diseases, Metabolic disorders

## Abstract

Hypertriglyceridemia (HTG) is an independent risk factor for atherosclerotic cardiovascular disease (ASCVD). One of the multiple origins of HTG alteration is impaired lipoprotein lipase (LPL) activity, which is an emerging target for HTG treatment. We hypothesised that early, even mild, alterations in LPL activity might result in an identifiable metabolomic signature. The aim of the present study was to assess whether a metabolic signature of altered LPL activity in a preclinical model can be identified in humans. A preclinical LPL-dependent model of HTG was developed using a single intraperitoneal injection of poloxamer 407 (P407) in male Wistar rats. A rat metabolomics signature was identified, which led to a predictive model developed using machine learning techniques. The predictive model was applied to 140 humans classified according to clinical guidelines as (1) normal, less than 1.7 mmol/L; (2) risk of HTG, above 1.7 mmol/L. Injection of P407 in rats induced HTG by effectively inhibiting plasma LPL activity. Significantly responsive metabolites (i.e. specific triacylglycerols, diacylglycerols, phosphatidylcholines, cholesterol esters and lysophospholipids) were used to generate a predictive model. Healthy human volunteers with the impaired predictive LPL signature had statistically higher levels of TG, TC, LDL and APOB than those without the impaired LPL signature. The application of predictive metabolomic models based on mechanistic preclinical research may be considered as a strategy to stratify subjects with HTG of different origins. This approach may be of interest for precision medicine and nutritional approaches.

## Introduction

Recent scientific evidence has shown that elevated plasma triglyceride (TG) concentrations, also known as hypertriglyceridemia (HTG), are an independent risk factor for atherosclerotic cardiovascular disease (ASCVD)^1–4^. HTG is the most common form of dyslipidaemia observed in the general population. It is estimated that approximately 30% of the European and American population have HTG (≥ 150 mg/dL or 1.7 mmol/L) and 1.7% have severe HTG (≥ 500 mg/dL or 5.7 mmol/L)^5^. Triglyceridemia can be defined as normal (< 150 mg/ dL or 1.7 mmol/L), borderline (150 to 199 mg/dL or 1.7 to 2.3 mmol/L) and high (200 to 499 mg/dL or 2.3 to 5.6 mmol/L) or very high/severe (≥ 500 mg/dL or 5.7 mmol/L), according to the European Society of Cardiology (ESC) and European Atherosclerosis Society (EAS) guidelines^6^. Although ASCVD risk is increased when TGs are > 1.7 mmol/L (150 mg/dL), the use of drugs to lower TG levels may only be considered in high-risk patients when TGs are > 2.3 mmol/L (200 mg/dL) and TGs cannot be lowered by lifestyle measures^7^.

HTG has a multifactorial origin, since there are different mechanisms that might lead to increased basal plasma TG levels. A combination of genetic factors, increased production or impaired clearance of triglyceride-rich lipoproteins (TRLs) are known to play a role in HTG^8^. Genetic causes include syndromes such as familial HTG (excess very low-density lipoprotein but normal cholesterol), familial combined hyperlipidaemia characterised by polymorphisms in apolipoprotein C-II (apoC-II) or apolipoprotein C-III (apoC-III); lipoprotein lipase (LPL) deficiency, apolipoprotein C-II deficiency, apolipoprotein AV (apoA-V) deficiency and dysbetalipoproteinemia^9^. Together with genetic factors, common non-communicable disorders of TRLs metabolism involve suboptimal lifestyle habits, including diets high in simple carbohydrates and/or saturated fat, excessive alcohol consumption, obesity, and sedentary behaviour among others^7^.

Several studies have shown that individuals at risk of ASCVD treated with statins present HTG despite lowering plasma LDL-C levels, resulting in a substantial residual in the risk of ASCVD^10^. Fibrate-based treatments have not shown a substantial reduction of HTG-associated ASCVD risk. Despite some experimental drugs based on increasing LPL activity have reported promising outcomes for HTG amelioration, no treatments have been approved to date due to early stages of development or adverse side effects^11^. Therefore, detection of early alterations in blood TG metabolism might provide an invaluable tool for stratifying the population according to HTG risk and subsequently personalising clinical or lifestyle-based strategies^12,13^.

In this context, LPL has been identified as a key regulator of TRL metabolism and is a target for pharmacological intervention against HTG^11,14^. In peripheral organs, LPL hydrolyses TGs packaged in lipoproteins, mainly chylomicrons and very-low-density lipoprotein (VLDL), to glycerol and free fatty acids for cellular internalisation and subsequent lipid storage and consumption. LPL activity is tightly regulated by several proteins, including several apolipoproteins and angiopoietin-like protein (ANGPTL)^15^. For example, loss-of-function mutations in APOC2 and APOCV inhibit LPL, resulting in severe HTG^16,17^. In contrast, ANGPTL3, ANGPTL4, ANGPTL8, and APOC3 inhibit LPL activity, thereby increasing TG levels^18^. Mice completely lacking LPL died within 18 h after birth and exhibited massive plasma HTG, with severe reductions in lipid droplets in many organs^19^. To date, more than 100 mutations in the LPL gene have been reported in patients with HTG^20^. Moreover, the expression of LPL in muscle cells and adipocytes is regulated by hormones (particularly insulin), nutritional status, and inflammation^21^.

Despite the relevance of early indicators of LPL dysfunction in the onset of HTG, tools to detect such dysregulation are lacking. In this context, preclinical models allow the characterisation and understanding of LPL-mediated HTG. Beyond the different approaches to generate preclinical models of HTG such as genetic variants, i.e. Zucker rats^22^, high-fat diets^23,24^, only the administration of the chemical compound poloxamer 407 (P407) can be considered as a non-genetic model of LPL-specific induction of HTG^25^. P407 is a non-ionic copolymer with different technological applications. Several studies have demonstrated that P407, when administered to experimental animals, inhibits the LPL activity and other lipases involved in blood TG clearance^25,26^. Thus, the P407-induced HTG model has been characterised in different rodent species (i.e. hamster, mouse or rat)^25,27–30^, concentrations (i.e. between 300 mg/kg^29^ and 1500 mg/kg bw^31^) and dosing (single^32^ or multiple doses^28^). Overall, it could be concluded that P407 administration is a well characterised model of LPL-specific HTG. In contrast to other approaches such as transgenic mice^19^, the chemical inhibition of LPL by P407 allows a fine control of the inhibition and a precise follow-up of the initial metabolic dysregulations caused by LPL underperformance.

In the present work, we hypothesised that early, even mild, alterations of LPL activity might result in an identifiable metabolic signature that can be captured using a combination of metabolomics and machine learning^33–35^. This signature may be used to identify individuals with early alterations of LPL function and therefore at risk of future HTG development or TRL clearance dysfunctions. The objective of the present work was to assess whether preclinical LPL dysregulation can be characterised by a metabolomics signature and whether this signature can be further identified in humans both at risk of HTG and healthy.

## Results

### Characterization and validation of the preclinical male Wistar rat model of P407-induced HTG

To characterize and validate the preclinical model of P407-induced HTG, biometric measurements, plasma parameters and liver biochemistry were determined (Table 1). No differences in body weight were observed between the groups. However, food consumption was higher in the P407 group. In addition, the P407 group showed a trend towards increased muscle and liver weights (Table 1). In relation to dyslipidaemia, plasma levels of TG and TC were significantly elevated in the P407 group. In addition, increased liver TC was confirmed in the P407 group without other significant changes in the liver (i.e. total lipids, TC, and phospholipids). The decrease in LPL activity is associated with an increase in plasma TG, consistent with the results in the P407 group. Several parameters related to carbohydrate dysfunction were also analysed to discard other related metabolic changes; no significant differences were found in glucose, non-esterified fatty acids (NEFAs) and insulin resistance parameters [i.e. Homeostatic Model Assessment Insulin Resistance (HOMA-IR), Homeostatic Model Assessment β-cells (HOMA-β) and Revised—Quantitative Insulin Sensitivity Check Index (R-QUICKI)]. Additionally, inflammation and oxidative stress were assessed with an increase in MCP-1 in the P407 group, and no significant differences were found in AST, ALT and 8-isoprostane.

**Table 1:** Characteristics of the preclinical model of P407-induced HTG.

		CON	P407	*p*-value
Biometric parameters	Initial body weight (g)	300.28 ± 4.09	300.33 ± 3.06	0.99
	Final body weight (g)	302.09 ± 3.85	304.57 ± 3.28	0.63
	Food intake (g)	18.49 ± 0.70	20.49 ± 0.48	0.03**
	RWAT weight (%)	3.52 ± 0.34	3.59 ± 0.27	0.87
	MWAT weight (%)	2.58 ± 0.21	2.54 ± 0.17	0.89
	Muscle weight (%)	1.78 ± 0.03	1.86 ± 0.04	0.09*
	Liver weight (%)	9.28 ± 0.54	10.38 ± 0.25	0.09*
	Cecum weight (%)	4.35 ± 0.18	4.15 ± 0.19	0.44
Plasma parameters	Glucose (mM)	132.45 ± 2.22	130.21 ± 4.73	0.68
	TG (mM)	92.84 ± 9.71	157.11 ± 18.26	0.01***
	TC (mM)	73.02 ± 2.58	81.10 ± 2.74	0.04**
	NEFAs (mM)	0.48 ± 0.04	0.50 ± 0.05	0.67
	Insulin (µg/L)	1.04 ± 0.18	0.91 ± 0.11	0.54
	HOMA-IR (au)	0.34 ± 0.06	0.30 ± 0.04	0.50
	HOMA-β (au)	5.34 ± 0.89	4.95 ± 0.64	0.75
	R-QUICKI (au)	0.59 ± 0.03	0.60 ± 0.04	0.89
	LPL activity (Δ nmol/mL·min)	5.37 ± 0.24	4.65 ± 0.20	0.04**
	MCP-1 (ng/mL)	9.78 ± 0.83	11.40 ± 0.44	0.10*
	AST (mU/mL)	1.25 ± 0.62	1.35 ± 0.67	0.29
	ALT (mU/mL)	2.57 ± 0.16	2.67 ± 0.17	0.82
Urine parameters	8-isoprostanes (ng/mL)	2.50 ± 0.78	2.66 ± 0.44	0.87
Liver biochemistry	Total lipids (mg/g)	41.48 ± 2.01	43.27 ± 3.18	0.64
	TC (mg/g)	1.31 ± 0.07	1.48 ± 0.13	0.30
	Phospholipids (mg/g)	11.56 ± 0.48	11.97 ± 0.78	0.66
	TG (mg/g)	3.70 ± 0.18	4.77 ± 0.43	0.04**

Results are presented as mean ± SEM (*n* = 10). Statistical comparisons between groups were made using Student’s *t* test. Some biometric parameters are presented as a percentage of g per kg of body weight to allow for proper comparison of parameters.

*RWAT* retroperitoneal white adipose tissue, *MWAT* mesenteric white adipose tissue, *TG* triglycerides, *TC* total cholesterol, *NEFAs* non-esterified fatty acids, *HOMA*-*IR* homeostatic model assessment insulin resistance, *HOMA*-*β* homeostatic model assessment β-cells, *R*-*QUICKI* revised—quantitative insulin sensitivity check index, *au* arbitrary units, *LPL activity* lipoprotein lipase activity (Δnmol/mL·min), *MCP*-*1* monocyte chemoattractant protein-1, *AST* aspartate aminotransferase (one unit of AST is the amount of enzyme that converts 1.0 µmole of glutamate per minute at pH 8.0 at 37 °C), *ALT*, alanine aminotransferase (one milliunit of ALT is defined as the amount of enzyme that produces 1.0 nmole of pyruvate per minute at 37 °C). Groups: CON, control HTG; *P407*, Poloxamer 407 induced HTG.

*Denotes *p* < 0.1 (trend), **p < 0.05 (significantly different) and ***p < 0.01 (highly significantly different) compared with control.

### Plasma metabolomic profiling and predictive modelling of the preclinical model of P407-induced HTG

Analysis of 126 key metabolites was included in the plasma metabolomic study of the preclinical model. An exploratory univariate analysis was performed to provide a preliminary list of altered metabolites prior to multivariate analysis (Table S1). Univariate analysis showed that 39 out of 126 metabolites were significantly altered between the groups after the MW test. After the BH correction, 11 specific lipids predominated among the 39 metabolites, including DGs, TGs, ChoEs, PCs and LPCs, which were mainly overrepresented in the P407 group. Unsupervised analysis (PCA) was performed to find intrinsic variation in plasma (Fig. S1). Although the groups were not initially separated by PCA, the OPLS-DA model was able to discriminate between the two groups (Fig. S2). The fitness and predictive accuracy of the OPLS-DA were determined by the values of R2X(_cum_) = 0.371, R2Y(_cum_) = 0.867, and Q2Y(_cum_) = 0.572 (Fig. S2d). Since the Q2Y value (0.572) was higher than the pQ2 value (0.02) and 0.5, we can conclude that the OPLS-DA model has a good predictive ability (Fig. S2b). These results indicate that it is possible to predict whether an animal has a hyperlipidaemic profile, based on the analysis of plasma lipidomics and metabolomics.

For the OPLS-DA model, the model equation is expressed as a linear combination of latent variables, providing a powerful framework for predictive modelling. The equation for predicting the response variable (Y) based on the predictor variables (X) can be formulated with the coefficients as:

$${\text{Y }} = b_{1} \cdot{\text{ Alanine }} + b_{2} \cdot{\text{ Proline }} + b_{2} \cdot{\text{ Valine }} + \, \ldots \, + b_{n} \cdot{\text{ TG}}\_{54}:{7 }( + RMSEE).$$

Here: Y is the response variable, *b*_*1*_*, b*_*2*_*, b*_*n*_*, …., b*_*n*_ are the coefficients, represented by the corresponding values in Table S2. Alanine, Proline, Valine, … ,TG_54:7 are the predictor variables (X), represented by the respective metabolite names. RMSEE (Root Mean Square Error of Estimation). This value indicates the average magnitude of the residual error in our model, and it is implicitly considered in the estimation of coefficients. Therefore, the equation implicitly accounts for the error in the model.

47 features were significantly altered with a VIP threshold of 1 (Table S1). PC 38:4 and DG 36:4 stand out with VIP > 2 and *q*-value < 0.01 in univariate analysis (Table S1). Finally, the metabolites presented in Table 2 are those with the highest statistical differences and predictive power in plasma that are related to glycerophospholipid, glycerolipid and steroid biosynthesis.

**Table 2:** Summary of the potentially predictive metabolites in the plasma of the preclinical model.

Metabolite	CON	P407	*p*-value	*q*-value	FC	VIP	Pathway
PC 38:4	14.02 ± 0.61	20.4 ± 0.88	< 0.01***	< 0.01***	1.46	2.21	Glycerophospholipid metabolism
LPC 18:0	50.40 ± 1.83	58.86 ± 1.39	< 0.01***	0.03**	1.17	1.85	
PC 36:4	13.76 ± 0.61	17.73 ± 0.7	< 0.01***	0.01**	1.29	1.83	
DG 36:4	1.53 ± 0.05	2.02 ± 0.03	< 0.01***	< 0.01***	1.33	2.03	Glycerolipid metabolism
DG 34:3	0.20 ± 0.01	0.31 ± 0.02	< 0.01***	< 0.01***	1.50	1.86	
DG 34:2	0.85 ± 0.04	1.05 ± 0.05	< 0.01***	0.04**	1.24	1.70	
TG 46:0	0.84 ± 0.05	1.16 ± 0.07	< 0.01***	0.04**	1.37	1.17	
ChoE (17:0)	0.13 ± 0.00	0.16 ± 0.01	< 0.01***	0.01**	1.28	1.75	Steroid biosynthesis
ChoE (18:0)	0.12 ± 0.01	0.18 ± 0.01	< 0.01***	0.03**	1.51	1.72	
ChoE (16:0)	2.02 ± 0.09	2.39 ± 0.07	< 0.01***	0.04**	1.18	1.61	
ChoE (18:1)	2.52 ± 0.11	3.54 ± 0.25	< 0.01***	0.04**	1.41	1.59	

Results are presented as the mean ± SEM (*n* = 10, group). Summary of univariant analysis includes *p*-value, *q*-value (pFDR) and FC (P407/CON); summary of the multivariate analysis is represented by VIP values of OPLS-DA; and metabolism pathway (KEGG). Using the criteria of pFDR < 0.05 and VIP > 1, 11 metabolites were selected as potential predictive metabolites.

*TG* triglyceride, *PC* phosphatidylcholine, *LPC* lysophospholipid, *DG* diacylglycerol, *ChoE* cholesterol ester.

*Denotes p < 0.1 (trend), **p < 0.05 (significantly different) and ***p < 0.01 (highly significantly different). Groups: CON, control HTG; P407, Poloxamer 407 induced HTG.

### Urine metabolomic profiling and predictive modelling of the preclinical model of P407-induced HTG

Forty-three metabolites were successfully detected in the NMR spectra of the urine metabolome of the preclinical model. An exploratory univariate analysis was performed to obtain a preliminary list of altered metabolites prior to multivariate analysis (Table S3). Moreover, 4 out of 43 metabolites were significantly different between the groups in urine after the MW test (i.e. trimethylamine N-oxide (TMAO), phenylacetylglycine (PAG), 2-deoxycytidine and leucine). Although, none of these metabolites remain altered after BH correction, the changes in magnitude were distinctive. Unsupervised analysis (PCA) was performed to find the intrinsic variation without observing a clear clustering (Fig. S3). Although both groups were partially separated in the OPLS-DA analysis (Fig. S4), the low Q2Y value (− 0.029) and the high pQ2 value in the permutation test suggest that this model is overfitted. Therefore, this urine metabolomic approach was discarded for further assessment in humans.

### Classification of healthy humans according to the preclinical prediction model

**Figure 1:**
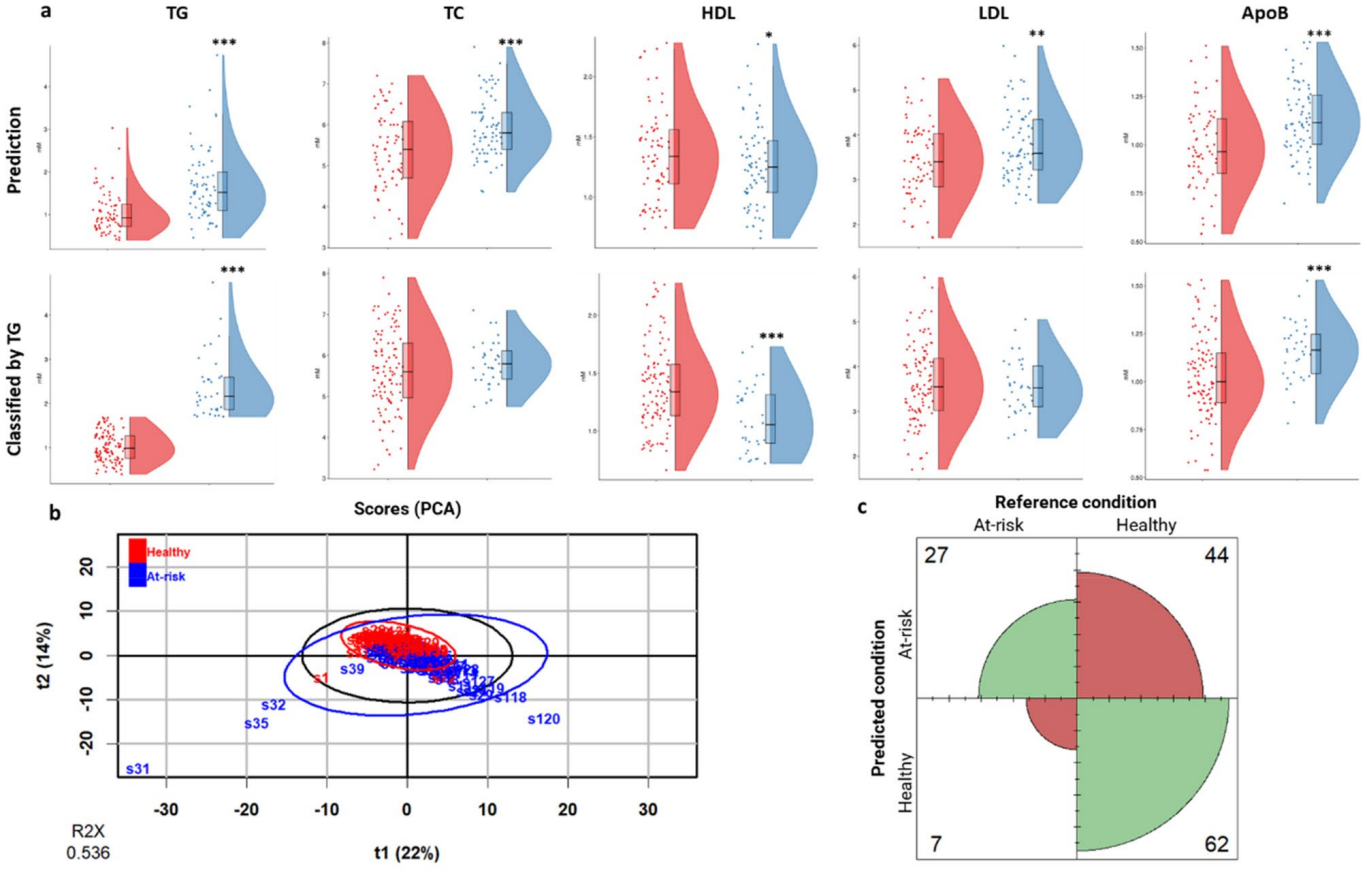
Evaluation of the classification of healthy individuals according to the preclinical predictive model. (**a**) Raincloud plot of TG, TC, HDL, LDL and APOB in healthy humans classified by the prediction model, TG and TC. *Denotes p < 0.1 (trend), **p < 0.05 (significantly different) and ***p < 0.01 (highly significantly different). Legend: red, healthy; blue, at-risk of LPL-mediated HTG. (**b**) Score plot (PCA) of human metabolomic data classified by the preclinical predictive model (healthy and at-risk of LPL-mediated HTG). (**c**) Confusion matrix for the classification by the reference condition TG. The confusion matrix is a table with 4 different combinations of predicted (preclinical model prediction) and reference values (TG). The positive condition is at-risk of LPL-mediated HTG. Legend: green colour, the prediction matches with the reference condition (true positive and true negative); red colour, the prediction does not match the reference condition (false positive and false negative). *TG* triglycerides, *TC* total cholesterol, *ApoB* apolipoprotein B-100.

The preclinical predictive model was subsequently applied to the metabolomic data from 140 volunteers. The projection resulted in 69 individuals classified as healthy and 71 individuals classified as at-risk of LPL-mediated HTG. The at-risk of LPL-mediated HTG group had statistically significantly higher levels of TG, TC, LDL and APOB and a tendency to decrease of HDL, while no differences were observed in age, BMI, BP, glucose levels and LPL (Fig. 1a and Table S4). Next, volunteers were divided in healthy and at-risk groups according to ESC/EAS guidelines. When the TG threshold of 1.7 mmol/L was used, statistically significant differences between healthy and at-risk individuals were detected for HDL and APOB (Fig. 1a and Table S5). Furthermore, the predictive model explains part of the variance in the data as two different clusters are observed in the PCA (Fig. 1b).

**Figure 2:**
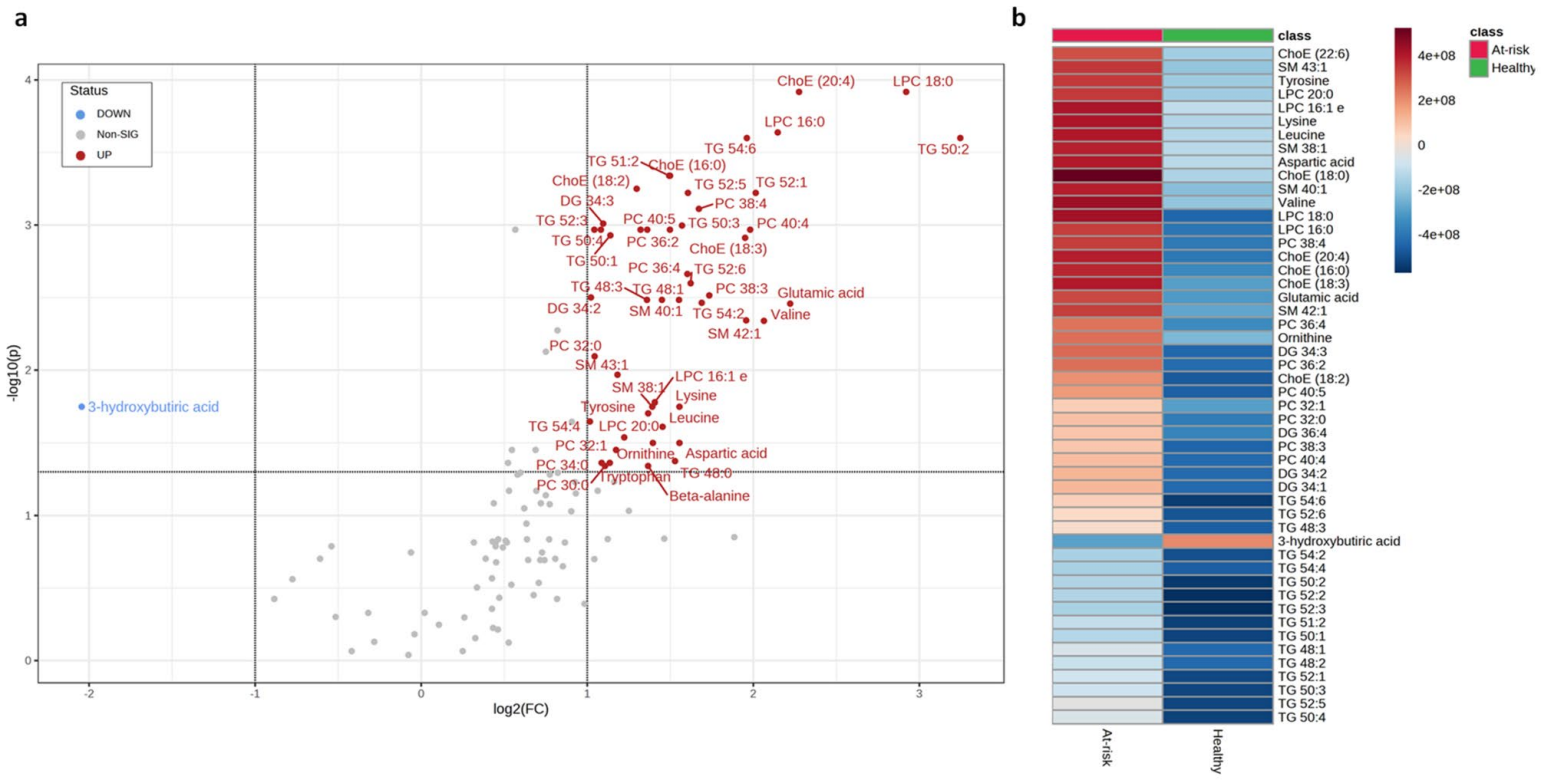
Differential metabolites of the plasma metabolome of healthy human population (according to ESC/EAS guidelines) classified as healthy and at-risk of LPL-mediated HTG (predictive model). (**a**) Volcano plot. The volcano plot combines the results of the fold change (FC) analysis on the x-axis and the false discovery rate (FDR) on the y-axis in a single graph, intuitively selecting significant features based on either biological significance, statistical significance, or both. The threshold values considered for the FC are values greater than or equal to 2 and for the FDR are values less than 0.1. (**b**) Heatmap of the top 50 metabolites in terms of FDR. A heatmap provides an intuitive visualization of a data table. Each column in the figure represents a group average; each row represents a differential metabolite expression value; blue indicates downregulation and brown indicates upregulation. These plots were generated by the online analysis software MetaboAnalyst 5.0^36^.

Next, a confusion matrix-based approach was used to compare the classifications obtained with the model with those obtained by applying the guideline thresholds. Thus, the confusion matrix comparing the model prediction with TG threshold classification presented good values (accuracy = 0.64; sensitivity = 0.79; and specificity = 0.58) (Fig. 1c). Our interest was focused on individuals classified as healthy by the guidelines but at-risk of LPL-mediated HTG by the model. In this line, we obtained 62 individuals who were predicted to be healthy by both the guidelines and the model, but 44 individuals who were classified as healthy by guidelines but at-risk of LPL-mediated HTG by the model. This last group is characterised by statistically higher levels of TG, TC, LDL and APOB than the first one (Table S6). Regarding the plasma metabolome, 67 metabolites related to lipid metabolism were significantly altered after the MW test and 46 of them remained altered after the BH correction (Table S7). Additionally, the volcano plot (Fig. 2a) showed 50 metabolites with FC threshold greater than or equal to 2 and FDR less than 0.1. Interestingly, only one metabolite was downregulated (3-hydroxybutyric acid) and the remaining metabolites were up-regulated at-risk of LPL-mediated HTG. This pattern is confirmed by the heatmap shown in the Fig. 2b.

To evaluate the metabolome of individuals classified as healthy by the guidelines but at-risk of LPL-mediated HTG by the model, multivariate analysis was performed to determine which metabolites were more important. Multivariate analysis showed a clustering tendency in PCA (Fig. S5) and clear differences in OPLS-DA (Fig. S6). As the Q2Y value (0.485) was higher than the pQ2 value (0.01), we can conclude that the OPLS-DA has a good predictive capacity (Fig. S6). According to the feature importance analysis, 54 features were significantly altered with a VIP threshold of 1 (Table S7). Finally, 39 metabolites stand out with VIP > 1 and *q*-value < 0.05 in univariate and multivariate analysis (Table S7), including several ChoEs, DGs, LPCs, PCs, SMs and TGs. These metabolites stand out as being distinctive among healthy individuals (according to the guidelines) but with early LPL-mediated HTG.

## Discussion

In this study, we report a slightly different preclinical male Wistar rat model of P407-induced HTG. The novelty relies in the dose of 150 mg of P407/kg, which is the lowest to date^25,27–29,31,32,37–39^. Thus, this dose was able to induce low levels of HTG and subsequent changes in the metabolic signature. The mechanism of action of P407 was confirmed by the effective inhibition of plasma LPL activity in the P407 group and parallel increases of plasma TG and TC levels^25^. These TG levels were the lowest reported in the literature for HTG models. In fact, TG levels were tenfold lower than in other studies with doses between 300 mg/kg and 1500 mg/kg^29,31^ and single or multiple injections^40–42^. Interestingly, these clinical values did not reach the characteristically high levels associated with pathological conditions requiring pharmacological treatment^7^. In addition, other risk factors associated with HTG such as carbohydrate dysfunction, inflammation and oxidative stress were assessed, and no differences were observed. This suggests that metabolomic changes result from early onset of LPL-mediated HTG.

The metabolomic changes result from the male Wistar rat preclinical model of P407-induced HTG consisted of increases of specific plasma lipids (e.g. DGs, PCs, ChoEs, LPCs and TGs). These findings were consistent with other studies^43–45^. For example, long-chain DGs and TGs were increased in our preclinical model as well as in other related studies^43,44^. In several studies, HTG is associated with increased ChoEs, since these metabolites are major players in fatty acid metabolism^43,45^. In particular, ChoE 17:0 and specific PCs (PC 36:4 and PC 38:4) have been reported as potential biomarkers in early HTG^43–49^, consistent with our findings. Overall, DGs, PCs, ChoEs, LPCs and TGs metabolites were key features of the predictive model based on the LPL dysregulation.

The metabolomic predictive model was further tested in healthy human volunteers with normal and at-risk TG levels (following guidelines). Using our predictive model, we identified a group of subjects with plasma TG levels of < 1.7 mmol/L (150 mg/dL), considered normal by guidelines^7^, but with the metabolomic profile indicator of early LPL dysfunction. Consistently, subjects predicted as at-risk of LPL-mediated HTG by our model presented higher levels of relevant clinical biomarkers, such as ApoB, TG, LDL-C or TC. Moreover, the differences in blood TC and LDL-C between healthy and at-risk of LPL-mediated HTG subjects were statistically significant when the classification was conducted with the predictive model but not according to TG guidelines. Overall, our results suggest that machine learning approaches can be used to better define indicators of early LPL dysfunction, complementing current guidelines that rely on thresholds that do not consider the mechanistic origin of disease.

Among the changes in subjects that presented the signature of early LPL dysfunction, certain metabolites have been highlighted such as DGs, PCs, ChoEs, LPCs and TGs. Additionally, a group of amino acids were up-regulated (aspartic acid, glutamic acid, leucine, lysine, tyrosine and valine). These results are consistent with emerging evidence suggesting that amino acids can have a role as modulators of lipid metabolism^50^. Thus, elevated levels of leucine, valine and lysine were significantly associated with an increased risk of developing HTG after 7 years (KORA S4 baseline study)^51^. Finally, only 3-hydroxybutyric acid, an intermediate metabolite of fatty acids, was found among the under-regulated metabolites^52^. Further mechanistical investigation would reveal whether a causal link between levels of these metabolites and LPL dysfunction exists.

In this regard, several authors have suggested that new biomarkers are needed to detect early deviations of relevant metabolic pathways. Thus, predictive biomarkers may allow the implementation of preventive strategies and personalised interventions^12,13,53,54^. For example, polygenetic risk /IBM^55^ or coronary artery calcium^56^ have been targeted to stratify early HTG without clinical ASCVD. Actually, one of the strategies for personalising treatments is based on stratifying the population according to metabotypes. This concept of grouping individuals into smaller, relatively homogeneous subgroups or clusters based on their metabolic phenotype has great potential for precision treatments^57^. Current approaches range from metabotypes obtained under fasting conditions to groups defined after meal challenges or dietary interventions^58^. For example, Van Bochove et al. described three distinct population subgroups according to the degree of dyslipidemia and different lipoprotein characteristics^59^. Interestingly, the different subgroups responded differently to fenofibrate treatment. Furthermore, they proposed that differences in lipoprotein profiles between subgroups may be due to different mechanisms leading to dyslipidaemia, i.e. impaired hepatic uptake in low and moderate dyslipidaemia and impaired systemic metabolism in the group with high dyslipidemia. In another study, Fiamoncini et al. described different metabolic responses to weight loss depending on the metabotype of the individual^58^. In this case, metabotypes were defined according to plasma fatty acid catabolites derived from lipolysis, fatty acid oxidation and ketogenesis. The authors suggested that lipid catabolites may allow early detection of the metabolic syndrome, as their blood concentrations vary depending on the interaction between insulin sensitivity and lipid metabolism. In both cases, differences in metabolic responses and metabolomic profiles have a mechanistic basis. Nevertheless, mechanistic evidence is usually lacking in metabotyping-based studies. In our approach, metabotypes were defined according to evidence generated in a preclinical study where a specific mechanism was targeted and used to define metabolomic profiles. Despite being a proof of concept, the approach presented here can be further extended to other causes of HTG or other metabolic alterations tightly related to human diseases.

This study has several limitations. It was designed as a proof of concept based on a secondary analysis, and no information on the long-term outcome of blood TG in these subjects is available. Therefore, more research is still needed to assess the clinical significance of our approach. Another important limitation is the use of the rat as the organism to generate the predictive model to be used with human data. Lipid metabolism is complex, and differences in LPL levels between rats and humans have been widely described^60^. Moreover, inter-individual variability in diet is not as controlled in humans as it is in laboratory animals. Despite the lipidome has been described as a reliable source of biomarkers in humans^61^, changes in fatty acid species resulting from different dietary habits might affect the interpretation of results. In our case, the use of a composite fingerprint rather than a single molecule as a biomarker allows to minimise the influence of single specific metabolites^62^. Nevertheless, the current design does not allow us to assess the impact of confounding factors such as diet, physical activity or metabolic conditions, which may affect a wider application of our model. Further research is needed to clarify the true clinical relevance of our prediction models and the limitations of their application.

To conclude, we have developed a pipeline that enables the development of predictive models of plasma TG levels by applying machine learning to metabolomics data obtained in preclinical research. In the current proof of concept, the pipeline was used to characterise a metabolomic fingerprint of early LPL dysregulation, characterised by elevated levels of DGs, PCs, ChoEs, LPCs and TGs. Subsequently, the model was used to identify healthy human volunteers that might be at risk of LPL-mediated HTG. Further research is needed to fully understand the clinical implications and significance of applying predictive machine learning models obtained from preclinical research to human data.

## Methods

### Preclinical model of P407-induced HTG

#### Experimental model

**Figure 3:**
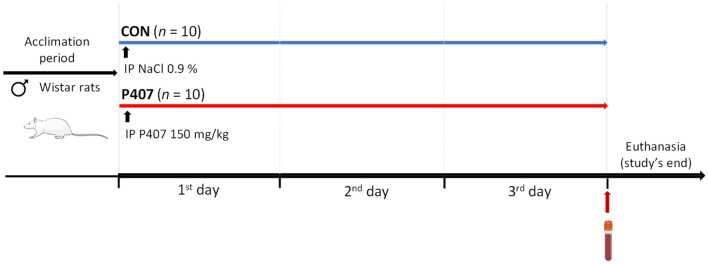
Schematic representation of the preclinical model of HTG induced by P407. The experimental model consisted of two groups of male Wistar rats (*n* = 10 animals per group). Each group received a single IP injection of 150 mg/kg of P407 and cold saline solution (NaCl 0.9%). Blood samples were collected at the end of the study. Groups: CON, control HTG; P407, Poloxamer 407 induced HTG. *IP* intraperitoneal, *P407* poloxamer 407.

Twenty 8-week-old male Wistar rats (Harlan Laboratories, Barcelona, Spain) were used to establish a model of P407-induced HTG. Our model consists of the administration of P407 as an inducer of HTG (Fig. 3). After an acclimatisation period, rodents were randomly divided into two experimental groups (*n* = 10): control group (CON) and P407-induced HTG group (P407). The treated group received P407 (Sigma-Aldrich, Madrid, Spain) by a single intraperitoneal (IP) injection of 80 mg/kg of body weight dissolved in cold saline solution (0.9% NaCl). Body weight was recorded on the day of IP injection and at the end of the study. Food consumption was estimated once. Food weight was recorded before the IP injection and 24 h later. Animals were housed individually under fully controlled conditions, including temperature (22 ± 2 °C), humidity (55 ± 5%) and light (12 h light–dark cycle and lights on at 9:00 a.m.). They were fed ad libitum with a standard rat diet (Teklad Global 18% Protein Rodent Diet 2014, Harlan, Barcelona, Spain). The Animal Ethics Committee of the University Rovira i Virgili (URV, Tarragona, Spain) approved all the procedures for the HTG model (code 10025). The experimental protocol followed the “Principles of Laboratory Care” and was performed in accordance with the Council Directive of the European Communities (86/609/EEC) and the ARRIVE (Animal Research: Reporting of In Vivo Experiments) guidelines.

#### Sample collection

At the end of the study, urine samples were collected using the hydrophobic sand method for metabolomics. This method is less stressful for the animals^63^. For each rat, 300 g of hydrophobic sand (LabSand, Coastline Global, Palo Alto, CA) was spread on the bottom of a plastic mouse cage. Urine was collected every half hour for 6 h. Urine was mixed with sodium azide (Sigma, St Louis, MO, USA) as a preservative at the end of the session. At the end of the study, the animals were euthanized by guillotine under anaesthesia (pentobarbital sodium, 50 mg/kg per body weight) after 7 h of fasting. Blood was collected using heparin as an anticoaguland and subsequently centrifuged at 3000 g for 15 min at 4 °C to recover plasma for metabolomics and other measures. Tissues (i.e. retroperitoneal white adipose tissue (RWAT), mesenteric white adipose tissue (MWAT), muscle, liver, and cecum) were quickly removed, weighted and snap frozen in liquid nitrogen. Samples were stored at − 80 °C until further analysis.

#### Plasma measurements

Enzymatic colorimetric kits were used for the determination of plasma total cholesterol (TC), TG, glucose (QCA, Barcelona, Spain) and non-esterified free fatty acids (NEFAs) (WAKO, Neuss, Germany). Circulating insulin levels were measured using a rat ELISA kit (Merck, Madrid, Spain). Monocyte chemoattractant protein-1 (MCP-1), as an inflammatory biomarker, was measured using the Rat MCP-1 Instant ELISA Kit (Invitrogen, Vienna, Austria). Oxidative stress was assessed by determination of plasma aspartate aminotransferase (AST) and alanine aminotransferase (ALT) activity (Sigma-Aldrich, St. Louis, USA) and urinary 8-isoprostane (Cayman chemical, Ann Arbor, MI, USA).

#### LPL activity

LPL enzymatic activity was assessed on plasma samples using a fluorescence method (LPL activity kit, Roar Biomedical, New York, USA). Aliquotes of 100 μL of plasma (1:4000 dilution) were incubated with 100 μL of pre-diluted substrate emulsion at 37 °C for 60 min according to the manufacturer’s recommendations. Determinations were performed in a 96-plate reader fluorimeter at 15-min intervals. The hydrolyzed triglycerides formed were measured at 370 nm excitation and 450 nm emission. The fluorescence intensity values of the samples were compared with the fluorescence intensity values of the standard curve applied to the same plate together with samples in each run. The increment between 60 and 15 min activity per mL and min (Δ nmol/mL·min) was used to calculate LPL activity. All measurements were made in duplicate and intra-and inter-assays coefficients of variation (CV) were less than 5%.

#### Liver lipids determinations

Liver lipids were extracted and quantified from an approximately 100 mg piece of frozen liver tissue using a method previously described in the literature^64^. Briefly, lipids were extracted with 1 ml hexane/isopropanol (3:2), degassed with gaseous nitrogen and allowed to stand overnight at room temperature under orbital agitation and protection from light. After extraction with 0.3 ml Na_2_SO_4_ (0.47 M), the lipid phase was dried with gaseous nitrogen. Total lipids were quantified gravimetrically before emulsification as described previously^65^. TG, TC and phospholipids were analysed using commercial enzymatic kits (QCA, Barcelona, Spain).

### Healthy volunteers

#### Samples and characteristics of human participants

A cross-sectional study was conducted with baseline data and plasma samples of 140 volunteers participating in previous studies registered under clinicaltrials.gov with references NCT02063477, NCT00511420, and NCT00502047. All protocols had been approved by the Clinical Research Ethical Committee of Hospital Universitari Sant Joan-Institut d’Investigació sanitaria Pere Virgili (Rf.13.05.30/5assN1, Rf.05.04.28/1al.leproj1 and Rf.03.09.2007), Reus, Catalonia, Spain. The protocols and trials had been conducted in accordance with the Helsinki Declaration and Good Clinical Practice Guidelines of the International Conference of Harmonization (GCP ICH). Volunteers had provided written consent.

Subjects were males and females between the ages of 43 and 65 years. At the time of sampling, all subjects were defined as healthy with no diagnosis of significant disease. To assess the risk of HTG, subjects were categorised according to their TG levels following the ESC/EAS guidelines for the management of dyslipidaemia^7^: (1) normal, less than 150 mg/dL or less than 1.7 mmol/L; (2) risk of HTG, above 150 mg/dL or above 1.7 mmol/L.

#### Sample analyses

All measurements have been described previously^66^. Body weight and body composition were obtained by a calibrated scale (Tanita SC 330-S; Tanita Corp., Barcelona, Spain). Height was measured using a wall-mounted stadiometer (Tanita Leicester Portable; Tanita Corp., Barcelona, Spain). Body mass index (BMI) was calculated as the ratio between measured weight (kg)/and the square of height (m). Blood pressure (BP) was measured twice by a physician using an automatic sphygmomanometer (OMRON HEM-907; Peroxfarma, Barcelona, Spain) after the subjects rested for 2–5 min in a seated position, with a 1-min interval measurements. Screening chemistries and hemograms were performed in the Hospital Universitari Sant Joan with appropriate clinical chemistry quality controls. Samples were stored at – 80 °C in the central laboratory’s Biobanc of Hospital Universitari Sant Joan—Eurecat (biobanc.reus@iispv.cat) until required for batch analyses. Plasma samples were profiled using classical determinations and plasma metabolomics as described below. Total cholesterol, HDL, LDL, TG, Apolipoprotein B-100 (ApoB) and fasting plasma glucose (FPG) were measured in plasma by standardized enzymatic automated methods in a PENTRA-400 autoanalyzer (ABX-Horiba Diagnostics, Montpellier, France). An enzymatic colorimetric kit was used for the determination of plasma lipoprotein lipase (LPL) enzymatic activity (Roar Biomedical, New York, USA).

### Preclinical and healthy population metabolomic approaches

#### Plasma metabolomics in male Wistar rats and healthy population

Plasma metabolites were analysed by gas Chromatography coupled with Quadrupole Time-of-Flight (GC-qTOF) in a preclinical model in male Wistar rats and healthy humans. For extraction, a protein precipitation extraction was performed by adding eight volumes of methanol:water (8:2) containing a mixture of internal standards (succinic acid-d_4_, myristic acid-d_27_, glycerol-^13^C_3_ and D-glucose-^13^C_6_) to the plasma samples. Then, the samples were mixed and incubated at 4 °C for 10 min, centrifuged at 21.420 g and the supernatant was evaporated to dryness before compound derivatization (metoximation and silylation). The derivatized compounds were analysed by GC-qTOF (model 7200 of Agilent, USA). Chromatographic separation was based on the Fiehn Method, using a J&W Scientific HP5-MS (30 m × 0.25 mm i.d.), 0.25 µm film capillary column and helium as carrier gas using an oven program from 60 °C to 325 °C. Ionization was done by electronic impact (EI), with electron energy of 70 eV and operated in full Scan mode. Metabolite identification was performed by matching their EI mass spectrum and retention time with the Fiehn metabolomics library (Agilent, Santa Clara, CA, USA) containing more than 1.400 metabolites. After putative identification of the metabolites, they were semi-quantified in terms of the internal standard response ratio.

Plasma lipids were analysed by Ultra High-Performance Liquid Chromatography coupled with Quadrupole Time-of-Flight (UHPLC-qTOF). For the extraction of hydrophobic lipids, a liquid–liquid extraction based on the Folch procedure was performed by adding four volumes of chloroform:methanol (2:1) containing an internal standard mixture (Lipidomic SPLASH®, Avanti Polar Lipids, Inc., Alabaster, AL, USA) to the plasma. Then, the samples were mixed and incubated at − 20 °C for 30 min. Afterwards, water with NaCl (0.8%) was added and the mixture was centrifuged at 21.420 g. The lower phase was recovered, evaporated to dryness and reconstituted with methanol:methyl-tert-butyl ether (9:1) and analysed by UHPLC-qTOF (model 6550 of Agilent, USA) in positive electrospray ionization mode. Chromatography consisted of an elution with a quaternary mobile phase containing water (A), methanol (B), and 2-propanol (C) and water with 200 mM ammonium formate and 2% formic acid (D). The gradient was as follows: 0–0.5 min, 40% A, 10% B, 45%C; 0.5–1.5 min, 37.8% A, 9.5% B, 47.7% C; 1.5–1.6 min, 28.7% A, 7.5% B, 58.8% C; 1.6–5 min, 26.8% A, 7% B, 61.2% C; 5–5.1 min, 13.6% A, 4% B, 77.4% C; 5.1–7.5 min, 11.4% A, 3.5% B, 80.1% C; 7.5–9 min, 11.4% A, 3.5% B, 80.1% C; 9–9.5 min, 95% C; 9.5–11.5 min, 95% C; 11.5–11.6 min, 40% A, 10% B, 45%C. The separation was carried out in a C18 column (Kinetex EVO C18 Column, 2.6 µm, 2.1 mm X 100 mm) at 60 °C that allows the sequential elution of the most hydrophobic lipids such as TG, diacylglycerols (DGs), phosphatidylcholines (PCs), cholesterol esters (ChoEs), lysophospholipids (LPCs) and sphingomyelins (SMs), among others. Identification of lipid species was performed by matching their accurate mass and tandem mass spectrum, when available, to Metlin-PCDL from Agilent containing more than 40,000 metabolites and lipids. In addition, the chromatographic behaviour of the pure standards of each family and literature information was used to ensure their putative identification: 1-Stearoyl-rac-glycerol (Sigma-Aldrich, Madrid, Spain), 1-Steraroyl-2-Hydroxy-sn-Glycero-3-Phosphocholine (Avanti Polar Lipids, Inc., Alabaster, AL, USA), 1,2-Dipalmitoyl-sn-glycero-3-phosphocholine (Avanti Polar Lipids, Inc., Alabaster, AL, USA), Sphingomyelin (Avanti Polar Lipids, Inc., Alabaster, AL, USA), 1,2-Dipalmitoyl-sn-glycero-3-phosphoethanolamine (Avanti Polar Lipids, Inc., Alabaster, AL, USA), 1,2-dioctadecanoyl-sn-glycerol (Avanti Polar Lipids, Inc., Alabaster, AL, USA), 1-Palmitoyl-2-oleoyl-3-linoleoyl-rac-glycerol (Sigma-Aldrich, Madrid, Spain) and Cholesteryl Palmitate (Sigma-Aldrich, Madrid, Spain). After putative lipid identification, lipids were semi-quantified in terms of internal standard response ratio using an internal standard for each lipid family.

A pooled matrix of samples was generated by taking a small volume of each experimental sample to serve as a technical replicate across the entire data set. As the study lasted several days, a data normalization step was performed to correct for variation resulting from inter-day instrument differences. Essentially, each compound was corrected in blocks of run days by quality controls, normalizing each data point proportionately.

### Urine metabolomics in male Wistar rats

Urine metabolites were analysed by proton Nuclear Magnetic Resonance (^1^H-NMR) in a preclinical model in male Wistar rats. The urine sample was mixed (1:1) with phosphate buffered saline containing with 3-(Trimethylsilyl)propionic-2,2,3,3-d_4_ acid sodium salt (TSP) (Sigma Aldrich) and placed in a 5 mm NMR tube for direct analysis by ^1^H-NMR. The ^1^H-NMR spectra were recorded at 300 K on an Avance III 600 spectrometer (Bruker®, Bremen, Germaney) operating at a proton frequency of 600.20 MHz using a 5 mm Broad Band Observe Probe (PBBO). Diluted urine aqueous samples were measured, and spectra information was recorded in procno 11 using a One-dimensional ^1^H pulse experiments which was carried out using nuclear Overhauser effect spectroscopy (NOESY). The NOESY presaturation sequence (RD–90°–t1–90°–tm–90° ACQ) was used to suppress the residual water peak, and the mixing time was set to 100 ms. A solvent presaturation with an irradiation power of 150 μW was applied during the recycling delay (RD = 5 s) and the mixing time. (noesypr1d pulse program in Bruker®, Bremen, Germany) to remove the residual water. The 90° pulse length was calibrated for each sample around 11 microsec. The spectral width was 9.6 kHz (16 ppm), and a total of 128 transients were collected into 64 k data points for each ^1^H spectrum. The exponential line broadening applied before the Fourier transformation was 0.3 Hz. The spectra in the frequency domain were manually phased and baseline corrected using TopSpin software (version 3.2, Bruker, Bremen, Germany). The data were normalized in two different ways, by probabilistic to avoid differences between samples due to different urine concentration, and by ERETIC. The acquired ^1^H-NMR was compared with pure compounds references from the AMIX spectra database of metabolic profiling (Bruker®, Bremen, Germany), HMDB, and Chenomx databases for metabolite identification. In addition, we assigned metabolites by ^1^H-^1^H homonuclear correlation (COSY and TOCSY) and ^1^H-^13^C heteronuclear (HSQC) 2D NMR experiments and by correlation with pure compounds run in-house. After pre-processing, specific ^1^H-NMR regions identified in the spectra were integrated using MATLAB scripts run in-house. The regions identified in the spectra were exported to an Excel spreadsheet to evaluate the robustness of the different ^1^H-NMR signals and to obtain the relative concentrations.

### Data analysis

#### Univariate statistical analysis

Statistical analysis was performed using R software (version 4.0.1, R Core Team 2021) and various libraries included in Bioconductor (version 3.11, Bioconductor project). Data were expressed as the mean ± standard error of the mean (SEM). Unpaired parametric t-test was used for individual statistical comparisons (*p* < 0.05). For metabolomic data, the z-score was calculated by subtracting the mean from the metabolite and then dividing the resulting number by the standard deviation of the dataset. This gives an indication of how far the metabolite is from the normal or typical value of the dataset. The Mann–Whitney (MW) test was used as the variables follow a non-parametric assumption. Adjustment of *p*-values for multiple comparisons was performed using the Benjamin-Hochberg (BH) correction with a false discovery rate (FDR) of 5%. The magnitude of the difference between populations is expressed as fold change (FC) relative to the control or healthy group^67^.

#### Multivariate modeling

Multivariate analysis, employing both an unsupervised method (Principal Component Analysis, PCA) and a supervised method (Orthogonal Partial Least-Squares Discriminant Analysis, OPLS-DA), was conducted to understand the global metabolic changes observed between the different groups. The analysis was performed using the ropls R package (version 1.19.16)^68^. The ropls package implements PCA and OPLS-DA approaches with the original, NIPALS-based versions of the algorithms^69,70^. The model equation is expressed as a linear combination of latent variables, providing a powerful framework for predictive modelling. The equation for predicting the response variable (Y) based on the predictor variables (X) can be formulated with the coefficients. This approach includes essential quality metrics such as R2Y and Q2Y^71^, permutation diagnostics^72^, computation of Variable Importance in the Projection (VIP) values^73^, and the evaluation of scores and orthogonal distances to detect outliers^74^. The package also provides various graphics for scores, loadings, predictions, diagnostics, and outlier detection.

The predictive performance of the data was assessed using the Q2Y parameter, calculated through cross-validation. Q2Y values are interpreted as follows: Q2Y < 0 indicates a model with no predictive ability, 0 < Q2Y < 0.5 indicates some predictive character, and Q2Y > 0.5 indicates good predictive ability^75^. Subsequently, VIP values were computed to select metabolites responsible for group differences. Metabolites meeting the criteria of qFDR < 0.05 and VIP > 1 were considered potential predictive metabolites, including statistical differences and predictive power.

**Figure 4:**
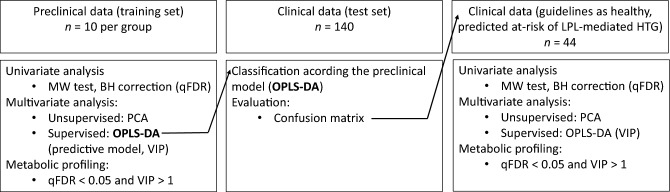
Data analysis pipeline. The experimental model consisted of two data sets: preclinical data (P407-induced HTG) and clinical data (healthy human cohort). *MW test* Mann–Whitney test, *BH correction* Benjamin-Hochberg correction, *PCA* principal component analysis, *OPLS*-*DA* orthogonal partial least-squares discriminant analysis, *qFDR* corrected p-value with false discovery rate, *VIP* variable importance in the projection.

The preclinical data (training set) was employed for model development, while the clinical data (test set) involved human samples for validation purposes. Thus, after obtaining the predictive model based on OPLS-DA in the preclinical model, we proceeded to assess the metabolomics of our human cohort, aiming to translate these findings to clinic. The prediction quality was evaluated by means of a confusion matrix along with its associated quality scores. Our subsequent focus centred on evaluating healthy individuals predicted to be at risk of HTG. These risk, not anticipated by the conventional guidelines, hold substantial interest due to their unique mechanism. Finally, the data analysis pipeline was summarized in Fig. 4.

### Approval for animal experiments

The study was conducted according to the guidelines of the Declaration of Helsinki and approved by the Ethics Committee of the University Rovira i Virgili (Tarragona, Spain) (protocol code 10025). The experimental protocol followed the “Principles of Laboratory Care” and was performed in accordance with the Council Directive of the European Communities (86/609/EEC) and the ARRIVE (Animal Research: Reporting of In Vivo Experiments) guidelines.

### Approval for human experiments

The protocols and trials had been conducted in accordance with the Helsinki Declaration and Good Clinical Practice Guidelines of the International Conference of Harmonization (GCP ICH). Informed consent was provided by all the volunteers. The study was conducted with baseline data and plasma samples of 140 volunteers participating in previous studies registered under clinicaltrials.gov with references NCT02063477, NCT00511420, and NCT00502047. All protocols had been approved by the Clinical Research Ethical Committee of Hospital Universitari Sant Joan-Institut d’Investigació sanitaria Pere Virgili (Rf.13.05.30/5assN1, Rf.05.04.28/1al.leproj1 and Rf.03.09.2007), Reus, Catalonia, Spain.

### Supplementary Information

Supplementary Information.

## Data Availability

The data presented in this study is available on request from the corresponding author. The data are not publicly available in the interest of performing more analysis for further publications together with more data.

## References

[1] Duran EK, Pradhan AD (2021). Triglyceride-rich lipoprotein remnants and cardiovascular disease. Clin. Chem..

[2] Castañer O (2020). Remnant cholesterol, Not LDL cholesterol, is associated with incident cardiovascular disease. J. Am. Coll. Cardiol..

[3] Laufs U, Parhofer KG, Ginsberg HN, Hegele RA (2020). Clinical review on triglycerides. Eur. Heart J..

[4] Nordestgaard BG (2016). Triglyceride-rich lipoproteins and atherosclerotic cardiovascular disease: New insights from epidemiology, genetics, and biology. Circ. Res..

[5] Hernandez P (2021). Clinical management of hypertriglyceridemia in the prevention of cardiovascular disease and pancreatitis. Curr. Atheroscler. Rep..

[6] Mach F (2020). 2019 ESC/EAS Guidelines for the management of dyslipidaemias: Lipid modification to reduce cardiovascular risk. Eur. Heart J..

[7] Mach F (2020). 2019 ESC/EAS Guidelines for the management of dyslipidaemias: lipid modification to reduce cardiovascular risk. Eur Heart J.

[8] Schaefer EJ, Geller AS, Endress G (2019). The biochemical and genetic diagnosis of lipid disorders. Curr. Opin. Lipidol..

[9] Rygiel K (2018). Hypertriglyceridemia - common causes, prevention and treatment strategies. Curr. Cardiol. Rev..

[10] Miller M (2008). Impact of triglyceride levels beyond low-density lipoprotein cholesterol after acute coronary syndrome in the PROVE IT-TIMI 22 trial. J. Am. Coll. Cardiol..

[11] Quispe R, Sweeney T, Varma B, Agarwala A, Michos ED (2022). Recent updates in hypertriglyceridemia management for cardiovascular disease prevention. Curr. Atheroscler. Rep..

[12] Hernandez-Baixauli J (2020). Detection of early disease risk factors associated with metabolic syndrome: A new era with the NMR metabolomics assessment. Nutrients.

[13] Tada H (2021). Personalized medicine for cardiovascular diseases. J. Hum. Genet..

[14] Fruchart J-C (2019). The selective peroxisome proliferator-activated receptor alpha modulator (SPPARMα) paradigm: Conceptual framework and therapeutic potential: A consensus statement from the International Atherosclerosis Society (IAS) and the Residual Risk Reduction Initi. Cardiovasc. Diabetol..

[15] Moon JH, Kim K, Choi SH (2022). Lipoprotein lipase: Is it a magic target for the treatment of hypertriglyceridemia. Endocrinol. Metab. (Seoul).

[16] Breckenridge WC, Little JA, Steiner G, Chow A, Poapst M (1978). Hypertriglyceridemia associated with deficiency of apolipoprotein C-II. N. Engl. J. Med..

[17] Pennacchio LA (2001). An apolipoprotein influencing triglycerides in humans and mice revealed by comparative sequencing. Science.

[18] Willer CJ (2013). Discovery and refinement of loci associated with lipid levels. Nat. Genet..

[19] Shi G (2021). Heterozygous lipoprotein lipase knockout mice exhibit impaired hematopoietic stem/progenitor cell compartment. Anim. Model. Exp. Med..

[20] Wu SA, Kersten S, Qi L (2021). Lipoprotein lipase and its regulators: An unfolding story. Trends Endocrinol. Metab..

[21] Packard CJ, Boren J, Taskinen M-R (2020). Causes and consequences of hypertriglyceridemia. Front. Endocrinol..

[22] Kasiske BL, O’Donnell MP, Keane WF (1992). The Zucker rat model of obesity, insulin resistance, hyperlipidemia, and renal injury. Hypertension.

[23] Gawronska-Szklarz B, Drozdzik M, Wojcicki J, Zakrzewski J (1994). Effect of experimental hyperlipidemia on the pharmacokinetics of digoxin. Acta Pol. Pharm..

[24] Sullivan MP, Cerda JJ, Robbins FL, Burgin CW, Beatty RJ (1993). The gerbil, hamster, and guinea pig as rodent models for hyperlipidemia. Lab. Anim. Sci..

[25] Johnston TP (2004). The P-407-induced murine model of dose-controlled hyperlipidemia and atherosclerosis: A review of findings to date. J. Cardiovasc. Pharmacol..

[26] Johnston TP (2010). Poloxamer 407 as a general lipase inhibitor: its implications in lipid metabolism and atheroma formation in C57BL/6 mice. J. Pharm. Pharmacol..

[27] Suárez-García S, Caimari A, del Bas JM, Suárez M, Arola L (2017). Serum lysophospholipid levels are altered in dyslipidemic hamsters. Sci. Rep..

[28] Korolenko TA (2016). Early-stage atherosclerosis in poloxamer 407-induced hyperlipidemic mice: Pathological features and changes in the lipid composition of serum lipoprotein fractions and subfractions. Lipids Health Dis..

[29] Johnston TP, Palmer WK (1997). The effect of pravastatin on hepatic 3-hydroxy-3-methylglutaryl CoA reductase obtained from poloxamer 407-induced hyperlipidemic rats. Pharmacotherapy.

[30] Blonder JM, Baird L, Fulfs JC, Rosenthal GJ (1999). Dose-dependent hyperlipidemia in rabbits following administration of poloxamer 407 gel. Life Sci..

[31] Tanko Y (2017). Effects of fermented ginger rhizome (*Zingiber*
*officinale*) and fenu greek (*Trigonella*
*foenum*-*graceum*) supplements on oxidative stress and lipid peroxidation biomarkers in poloxamer-407 induced -hyperlipidemic wistar rats. Niger. J. Physiol. Sci..

[32] Chaudhary HR, Brocks DR (2013). The single dose poloxamer 407 model of hyperlipidemia; systemic effects on lipids assessed using pharmacokinetic methods, and its effects on adipokines. J. Pharm. Pharm. Sci..

[33] Kaddurah-Daouk R, Kristal BS, Weinshilboum RM (2008). Metabolomics: A global biochemical approach to drug response and disease. Annu. Rev. Pharmacol. Toxicol..

[34] Ulaszewska MM (2019). Nutrimetabolomics: An integrative action for metabolomic analyses in human nutritional studies. Mol. Nutr. Food Res..

[35] Buergel T (2022). Metabolomic profiles predict individual multidisease outcomes. Nat. Med..

[36] Chong J (2018). MetaboAnalyst 4.0: Towards more transparent and integrative metabolomics analysis. Nucleic Acids Res..

[37] Johnston TP, Palmer WK (1993). Mechanism of poloxamer 407-induced hypertriglyceridemia in the rat. Biochem. Pharmacol..

[38] Korolenko TA (2012). The influence of repeated administration of poloxamer 407 on serum lipoproteins and protease activity in mouse liver and heart. Can. J. Physiol. Pharmacol..

[39] Blonder JM, Baird L, Fulfs JC, Rosenthal GJ (1999). Dose-dependent hyperlipidemia in rabbits following administration of poloxamer 407 gel. Life Sci..

[40] Joo IW, Ryu JH, Oh HJ (2010). The influence of Sam-Chil-Geun (*Panax*
*notoginseng*) on the serum lipid levels and inflammations of rats with hyperlipidemia induced by poloxamer-407. Yonsei Med. J..

[41] Yeom M (2018). Electroacupuncture ameliorates poloxamer 407-induced hyperlipidemia through suppressing hepatic SREBP-2 expression in rats. Life Sci..

[42] Hor S, Farsi E, Yam M, Nuyah N, Abdullah M (2011). Lipid-lowering effects of Coriolus *versicolor* extract in poloxamer 407-induced hypercholesterolaemic rats and high cholesterol-fed rats. J. Med. Plants Res..

[43] Ke C, Zhu X, Zhang Y, Shen Y (2018). Metabolomic characterization of hypertension and dyslipidemia. Metabolomics.

[44] Castro-Perez JM (2011). Identifying static and kinetic lipid phenotypes by high resolution UPLC–MS: Unraveling diet-induced changes in lipid homeostasis by coupling metabolomics and fluxomics. J. Proteome Res..

[45] Miao H (2015). Plasma lipidomics reveal profound perturbation of glycerophospholipids, fatty acids, and sphingolipids in diet-induced hyperlipidemia. Chem. Biol. Interact..

[46] Kwan HY (2013). Lipidomics identification of metabolic biomarkers in chemically induced hypertriglyceridemic mice. J. Proteome Res..

[47] Yin W (2012). Plasma lipid profiling across species for the identification of optimal animal models of human dyslipidemia. J. Lipid Res.

[48] Miao H (2016). Lipidomics biomarkers of diet-induced hyperlipidemia and its treatment with *Poria*
*cocos*. J. Agric. Food Chem..

[49] Du, H. et al. Proteomic and metabolomic analyses reveal the full spectrum of inflammatory and lipid metabolic abnormalities in dyslipidemia. 10.21203/rs.3.rs-135087/v1 (2020) 10.1002/bmc.5183 34058018

[50] Ma Q (2021). Dietary supplementation with aromatic amino acids decreased triglycerides and alleviated hepatic steatosis by stimulating bile acid synthesis in mice. Food Funct..

[51] Mook-Kanamori DO (2014). Increased amino acids levels and the risk of developing of hypertriglyceridemia in a 7-year follow-up. J. Endocrinol. Investig..

[52] Mierziak J, Burgberger M, Wojtasik W (2021). 3-hydroxybutyrate as a metabolite and a signal molecule regulating processes of living organisms. Biomolecules.

[53] van Ommen B, Keijer J, Heil SG, Kaput J (2009). Challenging homeostasis to define biomarkers for nutrition related health. Mol. Nutr. Food Res..

[54] Carneiro G, Radcenco AL, Evaristo J, Monnerat G (2019). Novel strategies for clinical investigation and biomarker discovery: A guide to applied metabolomics. Horm. Mol. Biol. Clin. Investig..

[55] Esteve-Luque V (2022). Polygenic risk of hypertriglyceridemia is modified by BMI. Int. J. Mol. Sci..

[56] Cainzos-Achirica M (2022). CAC for risk stratification among individuals with hypertriglyceridemia free of clinical atherosclerotic cardiovascular disease. JACC Cardiovasc. Imaging.

[57] Pigsborg K, Magkos F (2022). Metabotyping for precision nutrition and weight management: Hype or hope?. Curr. Nutr. Rep..

[58] Keijer J (2023). Omics biomarkers and an approach for their practical implementation to delineate health status for personalized nutrition strategies. Crit. Rev. Food Sci. Nutr..

[59] van Bochove K (2012). Clustering by plasma lipoprotein profile reveals two distinct subgroups with positive lipid response to fenofibrate therapy. PLoS One.

[60] Gordon SM (2015). A comparison of the mouse and human lipoproteome: Suitability of the mouse model for studies of human lipoproteins. J. Proteome Res..

[61] Hornemann T (2022). Lipidomics in biomarker research. Handb. Exp. Pharmacol..

[62] Califf RM (2018). Biomarker definitions and their applications. Exp. Biol. Med. (Maywood).

[63] Hoffman JF, Fan AX, Neuendorf EH, Vergara VB, Kalinich JF (2018). Hydrophobic sand versus metabolic cages: A comparison of urine collection methods for rats (*Rattus*
*norvegicus*). J. Am. Assoc. Lab. Anim. Sci..

[64] Caimari A, Del Bas JM, Crescenti A, Arola L (2013). Low doses of grape seed procyanidins reduce adiposity and improve the plasma lipid profile in hamsters. Int. J. Obes..

[65] Rodriguez-Sureda V, Peinado-Onsurbe J (2005). A procedure for measuring triacylglyceride and cholesterol content using a small amount of tissue. Anal. Biochem..

[66] Solà R (2012). Cocoa, hazelnuts, sterols and soluble fiber cream reduces lipids and inflammation biomarkers in hypertensive patients: A randomized controlled trial. PLoS One.

[67] Vinaixa M (2012). A guideline to univariate statistical analysis for LC/MS-based untargeted metabolomics-derived data. Metabolites.

[68] Thévenot EA, Roux A, Xu Y, Ezan E, Junot C (2015). Analysis of the human adult urinary metabolome variations with age, body mass index, and gender by implementing a comprehensive workflow for univariate and OPLS statistical analyses. J. Proteome Res..

[69] Wold S, Sjostrom M, Eriksson L (2001). PLS-regression: A basic tool of chemometrics. Chemom. Intell. Lab. Syst..

[70] Trygg J, Wold S (2002). Orthogonal projections to latent structures (O-PLS). J. Chemom..

[71] Mehmood T, Liland KH, Snipen L, Sæbø S (2012). A review of variable selection methods in partial least squares regression. Chemom. Intell. Lab. Syst..

[72] Szymanska E (2011). Double-check: Validation of diagnostic statistics for PLS-DA models in metabolomics studies. Metabolomics.

[73] Mevik B-H, Wehrens R (2007). The pls package: Principal component and partial least squares regression in R. J. Stat. Softw..

[74] Hubert M, Rousseeuw PJ, Vanden Branden K (2005). ROBPCA: A new approach to robust principal component analysis. Technometrics.

[75] Llorach-Asunción R, Jauregui O, Urpi-Sarda M, Andres-Lacueva C (2010). Methodological aspects for metabolome visualization and characterization: A metabolomic evaluation of the 24 h evolution of human urine after cocoa powder consumption. J. Pharm. Biomed. Anal..

