# Supplementary material for: Developing a model to predict the early risk of hypertriglyceridemia based on inhibiting lipoprotein lipase (LPL): a translational study
Source: Sci Rep. 2023 Dec 19;13:22646. doi: 10.1038/s41598-023-49277-w (PMC10730820; doi:10.1038/s41598-023-49277-w)
Supplement: Supplementary file 1 — Supplementary Information. [file 41598_2023_49277_MOESM1_ESM.docx]

**Supplementary Table 1. Summary of univariate/multivariate plasma analysis of the preclinical model in Wistar male rats.** The 126 metabolites are represented by the mean ± SEM (*n* = 10, group). Summary of univariate analysis includes *p*-value, *q*-value (pFDR) and FC (P407/CON). Summary of multivariate analysis is presented by VIP values of OPLS-DA. Metabolites are listed according to VIP values. * Denotes *p* < 0.1 (trend), ** *p* < 0.05 (significantly different) and *** *p* < 0.01 (highly significantly different). Groups: CON, control HTG; P407, Poloxamer 407 induced HTG. Groups: CON, control HTG; P407, Poloxamer 407 induced HTG. Abbreviations: DG, diacylglycerol; PC, phosphatidylcholine; ChoE, cholesteryl ester; LPC, lysophospholipid; TG, triglyceride; SM, sphingomyelin.

| Metabolite | CON | P407 | *p*-value | *q*-value | FC | VIP |
| --- | --- | --- | --- | --- | --- | --- |
| PC 38:4 | 14.02 ± 0.61 | 20.4 ± 0.88 | <0.01*** | <0.01*** | 1.46 | 2.21 |
| DG 36:4 | 1.53 ± 0.05 | 2.02 ± 0.03 | <0.01*** | <0.01*** | 1.33 | 2.03 |
| DG 34:3 | 0.2 ± 0.01 | 0.31 ± 0.02 | <0.01*** | <0.01*** | 1.50 | 1.86 |
| LPC 18:0 | 50.4 ± 1.83 | 58.86 ± 1.39 | <0.01*** | 0.03** | 1.17 | 1.85 |
| PC 36:4 | 13.76 ± 0.61 | 17.73 ± 0.7 | <0.01*** | 0.01** | 1.29 | 1.83 |
| ChoE (17:0) | 0.13 ± 0 | 0.16 ± 0.01 | <0.01*** | 0.01** | 1.28 | 1.75 |
| ChoE (18:0) | 0.12 ± 0.01 | 0.18 ± 0.01 | <0.01*** | 0.03** | 1.51 | 1.72 |
| DG 34:2 | 0.85 ± 0.04 | 1.05 ± 0.05 | <0.01*** | 0.04** | 1.24 | 1.70 |
| PC 36:2 | 11.88 ± 0.63 | 14.81 ± 0.67 | <0.01*** | 0.05* | 1.25 | 1.70 |
| TG 54:6 | 15.66 ± 1.84 | 32.62 ± 4.94 | <0.01*** | 0.07* | 2.08 | 1.65 |
| ChoE (16:0) | 2.02 ± 0.09 | 2.39 ± 0.07 | <0.01*** | 0.04** | 1.18 | 1.61 |
| ChoE (18:1) | 2.52 ± 0.11 | 3.54 ± 0.25 | <0.01*** | 0.04** | 1.41 | 1.59 |
| ChoE (20:4) | 65.98 ± 1.98 | 74.78 ± 2.06 | <0.01*** | 0.06* | 1.13 | 1.55 |
| PC 34:0 | 0.29 ± 0.01 | 0.32 ± 0.01 | 0.03** | 0.13 | 1.13 | 1.55 |
| TG 54:4 | 10.76 ± 1.6 | 25.48 ± 5.24 | 0.02** | 0.13 | 2.37 | 1.43 |
| TG 48:0 | 1.26 ± 0.11 | 2.19 ± 0.31 | 0.02** | 0.13 | 1.73 | 1.43 |
| PC 40:4 | 0.14 ± 0.01 | 0.18 ± 0.01 | 0.04** | 0.14 | 1.26 | 1.40 |
| TG 54:2 | 0.69 ± 0.07 | 1.36 ± 0.24 | 0.02** | 0.13 | 1.97 | 1.39 |
| TG 52:1 | 0.65 ± 0.06 | 1.22 ± 0.21 | 0.02** | 0.13 | 1.88 | 1.38 |
| TG 54:7 | 6.06 ± 0.92 | 12.67 ± 2.51 | 0.03** | 0.13 | 2.09 | 1.37 |
| TG 52:3 | 40.05 ± 6.14 | 81.4 ± 15.33 | 0.03** | 0.13 | 2.03 | 1.36 |
| TG 50:2 | 10.73 ± 1.62 | 22 ± 4.08 | 0.03** | 0.13 | 2.05 | 1.36 |
| TG 54:3 | 3.84 ± 0.45 | 7.75 ± 1.47 | 0.03** | 0.13 | 2.02 | 1.35 |
| TG 50:1 | 3.12 ± 0.45 | 6.46 ± 1.23 | 0.03** | 0.13 | 2.07 | 1.35 |
| TG 50:0 | 0.42 ± 0.03 | 0.74 ± 0.12 | 0.03** | 0.13 | 1.77 | 1.34 |
| PC 35:2 | 0.35 ± 0.02 | 0.42 ± 0.02 | 0.03** | 0.13 | 1.19 | 1.31 |
| TG 52:2 | 10.05 ± 1.59 | 20.73 ± 4.05 | 0.03** | 0.13 | 2.06 | 1.31 |
| TG 52:5 | 8.05 ± 1.17 | 16.74 ± 3.57 | 0.04** | 0.15 | 2.08 | 1.30 |
| TG 51:2 | 0.76 ± 0.07 | 1.34 ± 0.24 | 0.04** | 0.15 | 1.77 | 1.29 |
| ChoE (22:4) | 3.88 ± 0.24 | 4.49 ± 0.17 | 0.06* | 0.17 | 1.16 | 1.27 |
| TG 50:4 | 1.7 ± 0.27 | 3.21 ± 0.61 | 0.04** | 0.15 | 1.89 | 1.26 |
| Lactic acid | 7.79 ± 0.31 | 6.92 ± 0.39 | 0.09* | 0.25 | 0.89 | 1.26 |
| TG 52:6 | 1.25 ± 0.2 | 2.32 ± 0.45 | 0.05* | 0.16 | 1.87 | 1.26 |
| Lysine | 0.66 ± 0.04 | 0.76 ± 0.05 | 0.14 | 0.29 | 1.15 | 1.23 |
| Hydroxyproline | 0.87 ± 0.08 | 1.1 ± 0.1 | 0.09* | 0.24 | 1.27 | 1.22 |
| Malic acid | 0.38 ± 0.05 | 0.28 ± 0.02 | 0.07* | 0.19 | 0.74 | 1.22 |
| Alanine | 0.31 ± 0.04 | 0.39 ± 0.05 | 0.22 | 0.37 | 1.25 | 1.19 |
| TG 46:0 | 0.84 ± 0.05 | 1.16 ± 0.07 | <0.01*** | 0.04** | 1.37 | 1.17 |
| TG 48:2 | 1.4 ± 0.15 | 2.18 ± 0.33 | 0.05* | 0.16 | 1.56 | 1.16 |
| TG 50:3 | 5.61 ± 0.98 | 10 ± 1.87 | 0.06* | 0.17 | 1.78 | 1.16 |
| Alpha-ketoglutarate | 1.12 ± 0.05 | 1 ± 0.06 | 0.15 | 0.31 | 0.90 | 1.11 |
| ChoE (22:5) | 0.58 ± 0.07 | 0.71 ± 0.03 | 0.11 | 0.27 | 1.23 | 1.11 |
| Glucose | 0.71 ± 0.01 | 0.75 ± 0.03 | 0.26 | 0.41 | 1.06 | 1.07 |
| PC 38:3 | 0.78 ± 0.09 | 0.97 ± 0.08 | 0.13 | 0.29 | 1.24 | 1.04 |
| TG 48:3 | 0.48 ± 0.05 | 0.68 ± 0.09 | 0.08* | 0.22 | 1.42 | 1.03 |
| LPC 18:2 | 33.17 ± 1.34 | 29.66 ± 0.86 | 0.04** | 0.15 | 0.89 | 1.03 |
| Glycine | 2.02 ± 0.07 | 2.16 ± 0.09 | 0.22 | 0.37 | 1.07 | 1.00 |
| 2-hydroxyglutaric | 0.54 ± 0.05 | 0.44 ± 0.03 | 0.11 | 0.27 | 0.82 | 0.98 |
| Alpha-tocopherol | 0.66 ± 0.05 | 0.55 ± 0.05 | 0.14 | 0.29 | 0.84 | 0.98 |
| Beta-alanine | 0.05 ± 0 | 0.06 ± 0.01 | 0.11 | 0.27 | 1.24 | 0.96 |
| Glycerol | 3.68 ± 0.22 | 4.2 ± 0.32 | 0.20 | 0.35 | 1.14 | 0.94 |
| Methionine | 0.1 ± 0.01 | 0.12 ± 0.01 | 0.18 | 0.34 | 1.15 | 0.94 |
| Isoleucine | 0.12 ± 0.01 | 0.12 ± 0.01 | 0.94 | 0.97 | 1.01 | 0.93 |
| Leucine | 0.05 ± 0 | 0.04 ± 0 | 0.69 | 0.77 | 0.96 | 0.92 |
| TG 46:1 | 0.68 ± 0.07 | 0.84 ± 0.07 | 0.14 | 0.29 | 1.23 | 0.91 |
| LPC 18:0 e | 0.1 ± 0 | 0.09 ± 0 | 0.14 | 0.30 | 0.92 | 0.91 |
| LPC 20:0 | 0.29 ± 0.02 | 0.33 ± 0.02 | 0.15 | 0.31 | 1.12 | 0.91 |
| SM 36:1 | 1.09 ± 0.05 | 0.93 ± 0.05 | 0.04** | 0.15 | 0.85 | 0.90 |
| Tryptophan | 1.24 ± 0.06 | 1.29 ± 0.05 | 0.59 | 0.71 | 1.04 | 0.90 |
| TG 48:1 | 1.63 ± 0.16 | 2.18 ± 0.3 | 0.12 | 0.28 | 1.34 | 0.89 |
| LPC 16:0 e | 0.51 ± 0.02 | 0.47 ± 0.01 | 0.06* | 0.17 | 0.91 | 0.88 |
| PC 30:0 | 0.04 ± 0 | 0.04 ± 0 | 0.31 | 0.45 | 1.07 | 0.87 |
| Glyceric acid | 1.15 ± 0.1 | 0.96 ± 0.08 | 0.16 | 0.32 | 0.84 | 0.87 |
| LPC 16:1 e | 0.14 ± 0.01 | 0.13 ± 0 | 0.11 | 0.27 | 0.89 | 0.86 |
| PC 32:0 | 0.59 ± 0.02 | 0.62 ± 0.01 | 0.25 | 0.41 | 1.06 | 0.86 |
| PC 31:0 | 0.03 ± 0 | 0.04 ± 0 | 0.01** | 0.06* | 1.19 | 0.86 |
| Valine | 0.48 ± 0.02 | 0.46 ± 0.03 | 0.65 | 0.75 | 0.97 | 0.85 |
| 3-hydroxybutiric acid | 1.61 ± 0.08 | 1.44 ± 0.08 | 0.17 | 0.32 | 0.90 | 0.84 |
| Tyrosine | 0.59 ± 0.04 | 0.61 ± 0.03 | 0.68 | 0.76 | 1.03 | 0.84 |
| Fructose | 0.4 ± 0.02 | 0.34 ± 0.01 | 0.02** | 0.13 | 0.86 | 0.83 |
| ChoE (18:2) | 16.99 ± 0.99 | 18.25 ± 0.4 | 0.26 | 0.41 | 1.07 | 0.82 |
| Asparagine | 0.15 ± 0.02 | 0.18 ± 0.01 | 0.27 | 0.41 | 1.18 | 0.81 |
| TG 46:2 | 0.37 ± 0.03 | 0.5 ± 0.04 | 0.02** | 0.13 | 1.34 | 0.81 |
| LPC 18:1 | 13.89 ± 0.48 | 13.02 ± 0.42 | 0.19 | 0.35 | 0.94 | 0.80 |
| DG 34:1 | 1.02 ± 0.04 | 1.11 ± 0.06 | 0.22 | 0.37 | 1.09 | 0.80 |
| PC 33:0 | 0.03 ± 0 | 0.03 ± 0 | 1.00 | 1.00 | 1.00 | 0.66 |
| PC 40:5 | 0.16 ± 0.02 | 0.19 ± 0.02 | 0.41 | 0.57 | 1.15 | 0.66 |
| SM 39:1 | 0.14 ± 0.01 | 0.11 ± 0.01 | 0.08* | 0.23 | 0.78 | 0.66 |
| DG 36:2 | 1.26 ± 0.09 | 1.31 ± 0.05 | 0.69 | 0.77 | 1.03 | 0.65 |
| Ornithine | 2.4 ± 0.22 | 2.55 ± 0.21 | 0.64 | 0.75 | 1.06 | 0.65 |
| Glycolic acid | 2.99 ± 0.13 | 2.68 ± 0.16 | 0.14 | 0.30 | 0.90 | 0.64 |
| Pyruvic acid | 15.87 ± 1.01 | 14.88 ± 1.19 | 0.53 | 0.67 | 0.94 | 0.62 |
| SM 42:3 | 4.71 ± 0.28 | 4.15 ± 0.19 | 0.12 | 0.27 | 0.88 | 0.60 |
| Threonic acid | 2.08 ± 0.13 | 2.07 ± 0.08 | 0.93 | 0.97 | 0.99 | 0.57 |
| ChoE (20:2) | 0.79 ± 0.05 | 0.93 ± 0.06 | 0.08* | 0.23 | 1.18 | 0.57 |
| Glutamic acid | 0.1 ± 0.01 | 0.11 ± 0 | 0.30 | 0.44 | 1.09 | 0.56 |
| Histidine | 0.12 ± 0.01 | 0.14 ± 0.03 | 0.47 | 0.61 | 1.18 | 0.56 |
| Oleic acid | 1.6 ± 0.12 | 1.6 ± 0.08 | 0.97 | 0.98 | 1.00 | 0.55 |
| LPC 15:0 | 0.91 ± 0.04 | 0.87 ± 0.02 | 0.43 | 0.58 | 0.96 | 0.55 |
| ChoE (18:3) | 1.32 ± 0.08 | 1.4 ± 0.06 | 0.41 | 0.57 | 1.06 | 0.52 |
| Succinic acid | 0.64 ± 0.03 | 0.62 ± 0.03 | 0.60 | 0.71 | 0.97 | 0.51 |
| Serine | 0.26 ± 0.01 | 0.29 ± 0.03 | 0.29 | 0.44 | 1.13 | 0.49 |
| SM 35:1 | 0.16 ± 0.01 | 0.15 ± 0.01 | 0.21 | 0.36 | 0.90 | 0.45 |
| PC 33:1 | 0.06 ± 0 | 0.07 ± 0 | 0.20 | 0.36 | 1.13 | 0.45 |
| SM 36:2 | 0.4 ± 0.02 | 0.38 ± 0.02 | 0.49 | 0.63 | 0.96 | 0.44 |
| Proline | 0.24 ± 0.01 | 0.27 ± 0.01 | 0.19 | 0.35 | 1.09 | 0.40 |
| Urea | 2.18 ± 0.1 | 2.11 ± 0.1 | 0.60 | 0.71 | 0.96 | 0.40 |
| SM 38:1 | 0.47 ± 0.02 | 0.44 ± 0.02 | 0.29 | 0.44 | 0.93 | 0.40 |
| SM 32:1 | 0.22 ± 0.01 | 0.2 ± 0.01 | 0.30 | 0.44 | 0.94 | 0.38 |
| PC 32:2 | 0.19 ± 0.01 | 0.2 ± 0.01 | 0.46 | 0.61 | 1.06 | 0.37 |
| PC 34:1 | 3.59 ± 0.21 | 3.78 ± 0.18 | 0.51 | 0.65 | 1.05 | 0.36 |
| ChoE (16:1) | 0.56 ± 0.05 | 0.52 ± 0.05 | 0.56 | 0.69 | 0.93 | 0.36 |
| SM 42:2 | 9.81 ± 0.63 | 8.8 ± 0.46 | 0.21 | 0.37 | 0.90 | 0.35 |
| Ribose | 3.69 ± 0.23 | 4.03 ± 0.56 | 0.59 | 0.71 | 1.09 | 0.35 |
| SM 42:1 | 13.11 ± 0.53 | 12.31 ± 0.41 | 0.25 | 0.41 | 0.94 | 0.34 |
| SM 43:1 | 0.97 ± 0.04 | 0.92 ± 0.04 | 0.32 | 0.46 | 0.94 | 0.33 |
| PC 32:1 | 0.29 ± 0.03 | 0.26 ± 0.01 | 0.36 | 0.51 | 0.89 | 0.29 |
| SM 41:2 | 0.56 ± 0.03 | 0.58 ± 0.03 | 0.65 | 0.75 | 1.04 | 0.27 |
| LPC 16:0 | 78.98 ± 1.7 | 79.45 ± 1.23 | 0.83 | 0.88 | 1.01 | 0.26 |
| SM 33:1 | 0.31 ± 0.01 | 0.31 ± 0.01 | 0.95 | 0.97 | 1.00 | 0.26 |
| SM 41:1 | 3.54 ± 0.17 | 3.37 ± 0.13 | 0.46 | 0.61 | 0.95 | 0.23 |
| Citric acid | 3.87 ± 0.11 | 3.75 ± 0.12 | 0.48 | 0.62 | 0.97 | 0.20 |
| Cholesterol | 0.33 ± 0.01 | 0.3 ± 0.02 | 0.19 | 0.35 | 0.91 | 0.20 |
| SM 34:2 | 1.53 ± 0.08 | 1.44 ± 0.02 | 0.30 | 0.44 | 0.94 | 0.19 |
| PC 38:2 | 0.11 ± 0.01 | 0.12 ± 0.02 | 0.63 | 0.74 | 1.10 | 0.19 |
| SM 34:1 | 15.83 ± 0.76 | 15.09 ± 0.48 | 0.42 | 0.58 | 0.95 | 0.17 |
| ChoE (17:1) | 0.09 ± 0.01 | 0.1 ± 0.01 | 0.67 | 0.76 | 1.04 | 0.16 |
| Threonine | 1.18 ± 0.08 | 1.01 ± 0.05 | 0.11 | 0.27 | 0.86 | 0.15 |
| SM 40:1 | 3.3 ± 0.16 | 3.16 ± 0.13 | 0.52 | 0.65 | 0.96 | 0.14 |
| Fumaric acid | 0.86 ± 0.09 | 0.84 ± 0.1 | 0.92 | 0.97 | 0.98 | 0.14 |
| Aconitic acid | 0.01 ± 0 | 0.01 ± 0 | 1.00 | 1.00 | 1.00 | 0.11 |
| Phenylalanine | 0.58 ± 0.04 | 0.44 ± 0.04 | 0.03** | 0.14 | 0.77 | 0.10 |
| Aspartic acid | 0.55 ± 0.04 | 0.57 ± 0.04 | 0.73 | 0.80 | 1.04 | 0.07 |
| Glutamine | 1.19 ± 0.22 | 1.14 ± 0.2 | 0.85 | 0.90 | 0.95 | 0.06 |
| SM 40:2 | 0.66 ± 0.04 | 0.68 ± 0.03 | 0.76 | 0.82 | 1.02 | 0.04 |
| ChoE (22:6) | 2.03 ± 0.14 | 1.97 ± 0.12 | 0.74 | 0.80 | 0.97 | 0.01 |


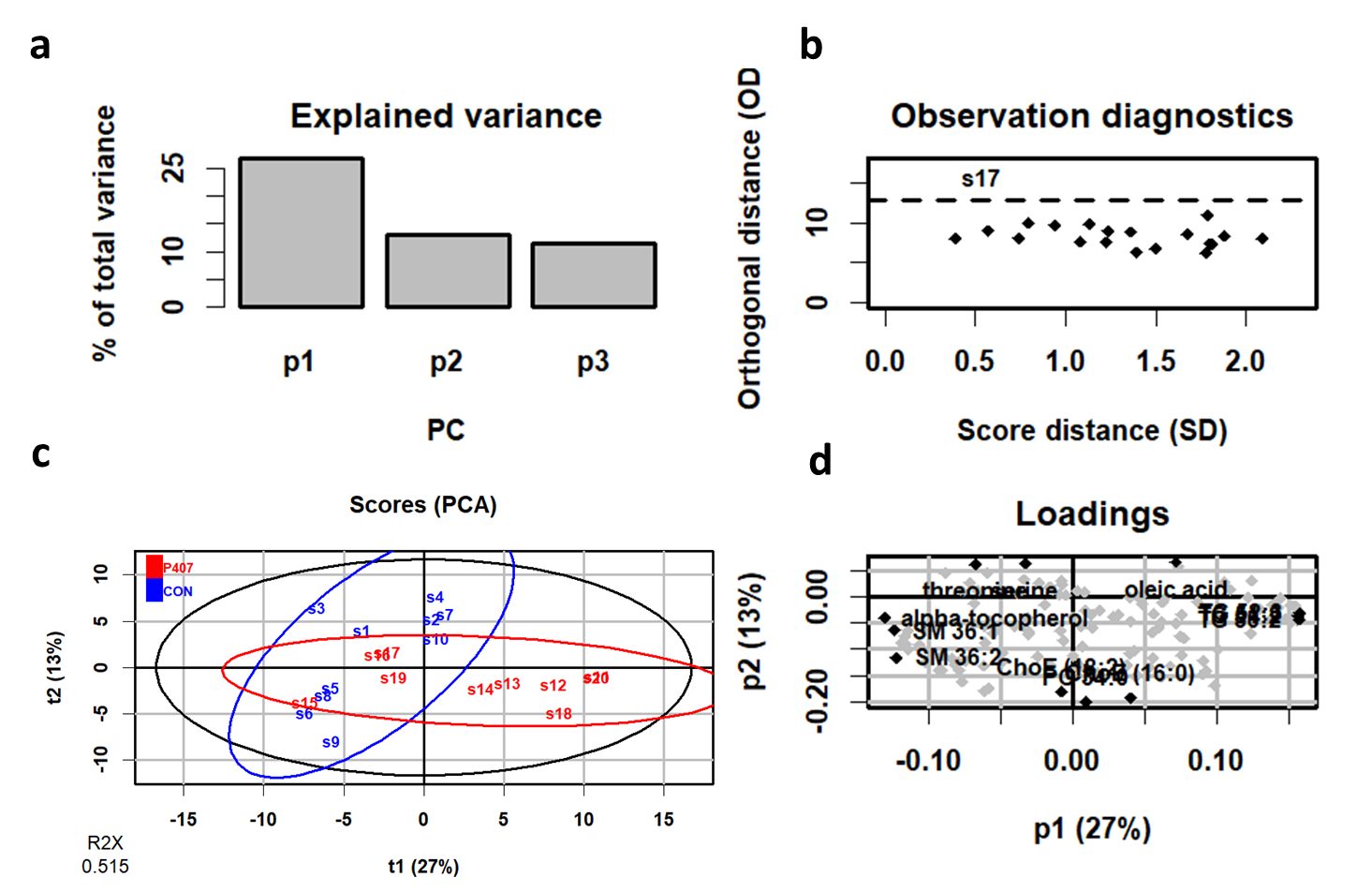


**Supplementary Figure 1. PCA summary plot for the plasma of the preclinical model** **in Wistar male rats.** (a) Explained variance. The scree plot suggests that 3 components may be sufficient to capture most of the variance. (b) Observation diagnostics. This plot shows the distances within and orthogonal to the projection plane; the names of the samples with a high value for at least one of the distances are given. (c) Score plot (PCA). The total variance explained is 40%: PC1 explains the 27% and the PC2 explains the 13%. (d) The variables with the most extreme values (positive and negative) for each loading are coloured black and labelled. Legend: blue, CON group; red, P407 group.

**Supplementary Figure 2. OPLS-DA summary plot for plasma of the preclinical model in Wistar male rats.** (a) Model overview. The plot here suggests that 2 components may be sufficient to capture most of the inertia (b) Significance diagnostics. The R2Y and Q2Y of the model are compared with the corresponding values obtained after random permutation of the y response. (c) Observation diagnostics. Indicates possible outliers. (d) X-score plot (OPLS-DA). The graph shows the number of components, the cumulative explained variance in X (R2X), in Y (R2Y), and the cumulative prediction power (Q2Y), the root mean square error of estimation (RMSEE), the predicted variation in Y (pre), and the orthogonal variance in Y (ort). Legend: Blue represents the CON group and red the P407 group.

**Supplementary Table 2. Coefficients of the predictor variables (OLPS-DA model for plasma in Wistar male rats).**

| Predictor variables (I) | Coefficients (I) | Predictor variables (II) | Coefficients (II) |
| --- | --- | --- | --- |
| **Alanine** | 0.0139117028 | **LPC 16:1 e** | -0.0190946569 |
| **Proline** | 0.0057509122 | **LPC 18:0** | 0.0319320995 |
| **Valine** | 0.0105542989 | **LPC 18:0 e** | -0.0200474328 |
| **Serine** | 0.0130068319 | **LPC 18:1** | -0.0149469051 |
| **Leucine** | 0.0108759464 | **LPC 18:2** | -0.0225170149 |
| **Threonine** | -0.0043935920 | **LPC 20:0** | 0.0167937361 |
| **Isoleucine** | 0.0112872433 | **PC 30:0** | 0.0160511513 |
| **Glycine** | 0.0134374147 | **PC 31:0** | 0.0178124534 |
| **Beta-alanine** | 0.0198563491 | **PC 32:0** | 0.0137691700 |
| **Aspartic acid** | 0.0044092255 | **PC 32:1** | -0.0043098115 |
| **Methionine** | 0.0147474134 | **PC 32:2** | 0.0082117366 |
| **Hydroxyproline** | 0.0189918620 | **PC 33:0** | 0.0087741095 |
| **Phenylalanine** | -0.0055752569 | **PC 33:1** | 0.0097059786 |
| **Glutamic acid** | 0.0117628177 | **PC 34:0** | 0.0222891315 |
| **Asparagine** | 0.0130216778 | **PC 34:1** | 0.0076614845 |
| **Glutamine** | -0.0021464986 | **PC 35:2** | 0.0237579685 |
| **Ornithine** | 0.0054485618 | **PC 36:2** | 0.0292554316 |
| **Histidine** | 0.0088270784 | **PC 36:4** | 0.0345699669 |
| **Lysine** | 0.0198816383 | **PC 38:2** | 0.0057213366 |
| **Tyrosine** | 0.0113656621 | **PC 38:3** | 0.0171346190 |
| **Tryptophan** | 0.0129384059 | **PC 38:4** | 0.0396757665 |
| **Pyruvic acid** | -0.0072002721 | **PC 40:4** | 0.0221817932 |
| **Lactic acid** | -0.0188417503 | **PC 40:5** | 0.0093300734 |
| **Glycolic acid** | -0.0163933058 | **SM 32:1** | -0.0140149957 |
| **3-hydroxybutiric acid** | -0.0157332056 | **SM 33:1** | 0.0022460412 |
| **Urea** | -0.0060925404 | **SM 34:1** | -0.0093152394 |
| **Glycerol** | 0.0147598941 | **SM 34:2** | -0.0080030301 |
| **Succinic acid** | -0.0062096638 | **SM 35:1** | -0.0128520525 |
| **Glyceric acid** | -0.0161067668 | **SM 36:1** | -0.0221983042 |
| **Fumaric acid** | -0.0011932335 | **SM 36:2** | -0.0085789191 |
| **Malic acid** | -0.0208742547 | **SM 38:1** | 0.0029028960 |
| **Threonic acid** | -0.0092229822 | **SM 39:1** | -0.0181507338 |
| **Alpha-ketoglutarate** | -0.0192362949 | **SM 40:1** | -0.0074965633 |
| **2-hydroxyglutaric** | -0.0182715323 | **SM 40:2** | 0.0032310267 |
| **Ribose** | -0.0009574493 | **SM 41:1** | -0.0086907206 |
| **Aconitic acid** | -0.0024919480 | **SM 41:2** | 0.0048742838 |
| **Citric acid** | -0.0081094713 | **SM 42:1** | -0.0131354080 |
| **Fructose** | -0.0139930938 | **SM 42:2** | -0.0142754758 |
| **Glucose** | 0.0133656925 | **SM 42:3** | -0.0177680716 |
| **Oleic acid** | 0.0086625096 | **SM 43:1** | -0.0111253129 |
| **Alpha-tocopherol** | -0.0166679209 | **TG 46:0** | 0.0220737853 |
| **Cholesterol** | 0.0027644096 | **TG 46:1** | 0.0168926176 |
| **ChoE (16:0)** | 0.0304155229 | **TG 46:2** | 0.0154115186 |
| **ChoE (16:1)** | -0.0065143167 | **TG 48:0** | 0.0270948726 |
| **ChoE (17:0)** | 0.0338773402 | **TG 48:1** | 0.0176241688 |
| **ChoE (17:1)** | 0.0046717433 | **TG 48:2** | 0.0221310976 |
| **ChoE (18:0)** | 0.0326773079 | **TG 48:3** | 0.0197446743 |
| **ChoE (18:1)** | 0.0322216753 | **TG 50:0** | 0.0248579545 |
| **ChoE (18:2)** | 0.0131756227 | **TG 50:1** | 0.0251593493 |
| **ChoE (18:3)** | 0.0096585823 | **TG 50:2** | 0.0252330213 |
| **ChoE (20:2)** | 0.0060455052 | **TG 50:3** | 0.0214235561 |
| **ChoE (20:4)** | 0.0286036539 | **TG 50:4** | 0.0229288540 |
| **ChoE (22:4)** | 0.0232762740 | **TG 51:2** | 0.0237015174 |
| **ChoE (22:5)** | 0.0181531774 | **TG 52:1** | 0.0254514819 |
| **ChoE (22:6)** | -0.0040345050 | **TG 52:2** | 0.0243881888 |
| **DG 34:1** | 0.0139497662 | **TG 52:3** | 0.0247693779 |
| **DG 34:2** | 0.0315234375 | **TG 52:5** | 0.0233217450 |
| **DG 34:3** | 0.0351110810 | **TG 52:6** | 0.0223093304 |
| **DG 36:2** | 0.0048063896 | **TG 54:2** | 0.0257023998 |
| **DG 36:4** | 0.0397463320 | **TG 54:3** | 0.0250637844 |
| **LPC 15:0** | -0.0091158438 | **TG 54:4** | 0.0260663582 |
| **LPC 16:0** | 0.0025775299 | **TG 54:6** | 0.0294310174 |
| **LPC 16:0 e** | -0.0211248353 | **TG 54:7** | 0.0245195758 |

**Supplementary Table 3**. **Urine univariate analysis of the preclinical model in Wistar male rats.** 43 metabolites are shown as mean ± SEM per group (*n* = 10, group). Summary of the univariate analysis includes *p*-value, *q*-value (pFDR) and FC (P407/CON). Metabolites are listed according to *q*-value. * Denotes *p* < 0.1 (trend), ** *p* < 0.05 (significantly different) and *** *p* < 0.01 (highly significantly different). Groups: CON, control HTG; P407, Poloxamer 407 induced HTG. Abbreviations: TMAO, trimethylamine N-oxide; PAG, phenylacetylglycine; DG, diacylglycerol; PC, phosphatidylcholine; ChoE, cholesteryl ester; LPC, lysophospholipid; TG, triglyceride; sphingomyelin, SM.

| Metabolite | CON | P407 | *p*-value | *q*-value | FC |
| --- | --- | --- | --- | --- | --- |
| TMAO | 2.31 ± 0.26 | 1.28 ± 0.09 | <0.01*** | 0.15 | 0.55 |
| PAG | 39.07 ± 3.42 | 63.78 ± 7.31 | 0.01** | 0.23 | 1.63 |
| 2-deoxycytidine | 1.89 ± 0.19 | 1.36 ± 0.11 | 0.03** | 0.35 | 0.72 |
| Leucine | 11.97 ± 1.03 | 9.23 ± 0.38 | 0.03** | 0.35 | 0.77 |
| 3-hydroxyisovalerate | 3.38 ± 0.15 | 2.90 ± 0.16 | 0.06* | 0.55 | 0.86 |
| Betaine | 25.78 ± 2.25 | 21.36 ± 0.62 | 0.09* | 0.62 | 0.83 |
| HPPA sulfate | 6.98 ± 1.77 | 14.91 ± 3.68 | 0.10* | 0.62 | 2.14 |
| o-Coumaric acid | 3.48 ± 0.58 | 4.99 ± 0.75 | 0.13 | 0.67 | 1.44 |
| Creatinine | 126.44 ± 6.22 | 114.02 ± 4.64 | 0.13 | 0.67 | 0.90 |
| Trimethylamine | 0.91 ± 0.12 | 1.37 ± 0.26 | 0.18 | 0.79 | 1.50 |
| Malate | 1.92 ± 0.12 | 2.84 ± 0.65 | 0.20 | 0.79 | 1.48 |
| Tyrosine | 14.15 ± 3.39 | 21.75 ± 4.56 | 0.21 | 0.79 | 1.54 |
| N,N-Dimethylglycine | 6.09 ± 1.19 | 4.53 ± 0.52 | 0.26 | 0.79 | 0.74 |
| 2-Hydroxyisobutyrate | 0.004 ± 0.001 | 0.003 ± 0.0007 | 0.27 | 0.79 | 0.62 |
| Formate | 1.60 ± 0.25 | 2.14 ± 0.39 | 0.27 | 0.79 | 1.34 |
| Glycine | 10.24 ± 0.78 | 9.21 ± 0.50 | 0.28 | 0.79 | 0.90 |
| 4-PY | 2.98 ± 0.55 | 2.31 ± 0.34 | 0.32 | 0.81 | 0.78 |
| 3-HPPA | 9.90 ± 2.38 | 14.22 ± 3.47 | 0.35 | 0.81 | 1.44 |
| Fumarate | 3.78 ± 0.52 | 3.10 ± 0.39 | 0.36 | 0.81 | 0.82 |
| Allantoin | 230.44 ± 5.54 | 222.16 ± 7.86 | 0.41 | 0.81 | 0.96 |
| Sarcosine | 3.99 ± 0.36 | 4.36 ± 0.26 | 0.43 | 0.87 | 1.09 |
| Indoxyl Sulphate | 7.50 ± 0.73 | 8.39 ± 1.01 | 0.48 | 0.87 | 1.12 |
| Tryptophan | 7.52 ± 0.74 | 8.40 ± 1.02 | 0.49 | 0.87 | 1.12 |
| Alanine | 3.97 ± 0.28 | 3.74 ± 0.16 | 0.50 | 0.87 | 0.94 |
| Methylamine | 5.17 ± 0.21 | 4.97 ± 0.20 | 0.52 | 0.87 | 0.96 |
| N-acetylglycoproteins | 73.29 ± 8.32 | 66.48 ± 5.35 | 0.54 | 0.87 | 0.91 |
| 2-Oxoglutarate | 142.06 ± 13.08 | 129.55 ± 15.72 | 0.56 | 0.87 | 0.91 |
| 3-methyl-2-oxovalerate | 4.20 ± 0.39 | 3.95 ± 0.20 | 0.59 | 0.87 | 0.94 |
| Hippurate | 267.88 ± 27.15 | 245.29 ± 32.31 | 0.60 | 0.87 | 0.92 |
| Acetate | 4.74 ± 0.58 | 5.07 ± 0.39 | 0.64 | 0.87 | 1.07 |
| 1-methylnicotinamide | 0.02 ± 0.01 | 0.03 ± 0.01 | 0.65 | 0.87 | 1.24 |
| Pseudouridine | 10.38 ± 0.73 | 10.14 ± 0.50 | 0.79 | 0.88 | 0.98 |
| α-hydroxyhippurate | 1.09 ± 0.11 | 1.12 ± 0.09 | 0.82 | 0.88 | 1.03 |
| Succinate | 43.05 ± 3.57 | 43.96 ± 2.80 | 0.84 | 0.95 | 1.02 |
| Taurine | 417.08 ± 39.53 | 428.39 ± 40.02 | 0.84 | 0.95 | 1.03 |
| Dimethylamine | 49.30 ± 2.54 | 48.57 ± 2.33 | 0.86 | 0.95 | 0.99 |
| N-Acetylglycine | 29.61 ± 2.69 | 29.02 ± 1.87 | 0.87 | 0.95 | 0.98 |
| Citrate | 252.17 ± 20.74 | 258.04 ± 28.6 | 0.88 | 0.95 | 1.02 |
| Fucose | 10.19 ± 0.38 | 10.26 ± 0.54 | 0.92 | 0.95 | 1.01 |
| NAD+ | 0.30 ± 0.04 | 0.30 ± 0.04 | 0.92 | 0.95 | 0.98 |
| Valine | 1.11 ± 0.12 | 1.10 ± 0.02 | 0.93 | 0.95 | 0.99 |
| N6-Acetyllysine | 16.84 ± 0.91 | 16.72 ± 1.02 | 0.93 | 0.95 | 0.99 |
| Lactate | 10.19 ± 0.97 | 10.17 ± 0.76 | 0.99 | 0.95 | 1.00 |

**Supplementary Figure 3. PCA summary plot for the urine of the preclinical model in Wistar male rats.** (a) Explained variance. The scree plot suggests that 4 components may be sufficient to capture most of the variance. (b) Observation diagnostics. This plot shows the distances within and orthogonal to the projection plane; the names of the samples with a high value for at least one of the distances are given. (c) Score plot (PCA). The total variance explained is 37%: PC1 explains the 21% and the PC2 explains the 16%. (d) The variables with the most extreme values (positive and negative) for each loading are coloured black and labelled. Legend: blue, CON group; red, P407 group.

**Supplementary Figure 4. OPLS-DA summary plot for the urine of the preclinical model in Wistar male rats.** (a) Model overview. The plot here suggests that 2 components may be sufficient to capture most of the inertia (b) Diagnostic significance. The R2Y and Q2Y of the model are compared with the corresponding values obtained after random permutation of the y-response. (c) Observation diagnostics. Indicates possible outliers. (d) X-score plot (OPLS-DA). The graph shows the number of components, the cumulative explained variance in X (R2X), in Y (R2Y), and the cumulative prediction power (Q2Y), the root mean square error of estimation (RMSEE), the predicted variation in Y (pre), and the orthogonal variance in Y (ort). Legend: blue, CON group; red, P407 group.

**Supplementary Table 4**. **Characteristics of the human population classified by the preclinical predictive model**. Results are presented as the mean ± SEM. Statistical comparisons between groups were performed using *t*-student. * Denotes p < 0.1 (trend), ** p < 0.05 (significantly different) and *** p < 0.01 (highly significantly different). Abbreviations: BMI, body mass index; SBP, systolic blood pressure; DBP, diastolic blood pressure; FPG, fasting plasma glucose; TG, triglycerides; TC, total cholesterol; LDL, low-density lipoprotein cholesterol; HDL, high-density lipoprotein cholesterol; APOB, apolipoprotein B-100; LPL activity, lipoprotein lipase activity (Δ nmol/mL·min).

| Prediction | Healthy  (*n* = 69) | At-risk of LPL-mediated HTG  (*n* = 71) | *p*-value |
| --- | --- | --- | --- |
| Age | 51.84 ± 1.73 | 50.15 ± 1.67 | 0.48 |
| BMI | 26.33 ± 0.35 | 26.69 ± 0.27 | 0.43 |
| SBP (mmHg) | 137.68 ± 1.9 | 133.27 ± 2 | 0.11 |
| DBP (mmHg) | 84.01 ± 1.2 | 83.18 ± 1.12 | 0.61 |
| FPG (mM) | 5.42 ± 0.07 | 5.57 ± 0.1 | 0.24 |
| TG (mM) | 1.04 ± 0.06 | 1.67 ± 0.1 | <0.01*** |
| TC (mM) | 5.37 ± 0.11 | 5.95 ± 0.09 | <0.01*** |
| LDL (mM) | 3.42 ± 0.1 | 3.8 ± 0.09 | 0.01** |
| HDL (mM) | 1.36 ± 0.05 | 1.27 ± 0.04 | 0.10* |
| APOB (mM) | 0.98 ± 0.03 | 1.14 ± 0.02 | <0.01*** |
| LPL activity (Δ) | 1.46 ± 0.01 | 1.45 ± 0.01 | 0.29 |

**Supplementary Table 5**. **Characteristics of the human population classified by TG levels (according to guidelines)**. Results are presented as the mean ± SEM. Statistical comparisons between groups were performed using *t*-student. * Denotes p < 0.1 (trend), ** p < 0.05 (significantly different) and *** p < 0.01 (highly significantly different). Abbreviations: BMI, body mass index; SBP, systolic blood pressure; DBP, diastolic blood pressure; FPG, fasting plasma glucose; TG, triglycerides; TC, total cholesterol; LDL, low-density lipoprotein cholesterol; HDL, high-density lipoprotein cholesterol; APOB, apolipoprotein B-100; LPL activity, lipoprotein lipase activity (Δ nmol/mL·min).

| TG levels | Healthy  (*n* = 106) | At-risk TG levels  (*n* = 34) | *p*-value |
| --- | --- | --- | --- |
| Age | 50.04 ± 1.43 | 53.94 ± 2.07 | 0.13 |
| BMI | 26.44 ± 0.25 | 26.76 ± 0.46 | 0.55 |
| SBP (mmHg) | 135.79 ± 1.59 | 134.35 ± 2.92 | 0.67 |
| DBP (mmHg) | 83.35 ± 0.96 | 84.31 ± 1.53 | 0.60 |
| FPG (mM) | 5.42 ± 0.05 | 5.73 ± 0.19 | 0.11 |
| TG (mM) | 1.03 ± 0.03 | 2.39 ± 0.12 | <0.01*** |
| TC (mM) | 5.61 ± 0.09 | 5.81 ± 0.09 | 0.13 |
| LDL (mM) | 3.64 ± 0.09 | 3.53 ± 0.11 | 0.46 |
| HDL (mM) | 1.38 ± 0.04 | 1.1 ± 0.05 | <0.01*** |
| APOB (mM) | 1.03 ± 0.02 | 1.16 ± 0.03 | <0.01*** |
| LPL activity (Δ) | 1.45 ± 0.01 | 1.45 ± 0.01 | 0.67 |

**Supplementary Table 6**. **Characteristics of the healthy human population (according to TG guidelines) classified as healthy and at-risk of LPL-mediated HTG (predictive model)**. Results are presented as the mean ± SEM. Statistical comparisons between groups were performed using *t*-student. * Denotes p < 0.1 (tendency), ** p < 0.05 (significantly different) and *** p < 0.01 (high significantly different). Abbreviations: BMI, body mass index; SBP, systolic blood pressure; DBP, diastolic blood pressure; FPG, fasting plasma glucose; TG, triglycerides; TC, total cholesterol; LDL, low-density lipoprotein cholesterol; HDL, high-density lipoprotein cholesterol; APOB, apolipoprotein B-100; LPL activity, lipoprotein lipase activity (Δ nmol/mL·min).

|  | Predicted healthy  (*n* = 62) | Predicted at-risk of LPL-mediated HTG  (*n* = 44) | *p*-value |
| --- | --- | --- | --- |
| Age | 51.02 ± 1.84 | 48.66 ± 2.27 | 0.42 |
| BMI | 26.4 ± 0.38 | 26.5 ± 0.29 | 0.83 |
| SBP (mmHg) | 136.95 ± 2.03 | 134.16 ± 2.55 | 0.39 |
| DBP (mmHg) | 83.06 ± 1.27 | 83.77 ± 1.49 | 0.72 |
| FPG (mM) | 5.38 ± 0.07 | 5.46 ± 0.09 | 0.49 |
| TG (mM) | 0.92 ± 0.04 | 1.19 ± 0.05 | <0.01*** |
| TC (mM) | 5.34 ± 0.12 | 6 ± 0.13 | <0.01*** |
| LDL (mM) | 3.42 ± 0.1 | 3.94 ± 0.13 | <0.01*** |
| HDL (mM) | 1.4 ± 0.05 | 1.36 ± 0.05 | 0.65 |
| APOB (mM) | 0.97 ± 0.03 | 1.11 ± 0.03 | <0.01*** |
| LPL activity (Δ) | 4.37 ± 0.17 | 4.35 ± 0.15 | 0.61 |

**Supplementary Table 7**. **Summary of univariate/multivariate plasma analysis of the healthy human population (according to TG guidelines) classified as healthy and at-risk** **of LPL-mediated HTG (predictive model)**. 126 metabolites are shown as the mean ± SEM (*n* = 10, group). Summary of univariate analysis includes *p*-value, *q*-value (pFDR) and FC (at-risk of LPL-mediated HTG /healthy). Summary of the multivariate analysis is presented by VIP values of OPLS-DA. Metabolites are listed according to VIP values. * Denotes *p* < 0.1 (trend), ** *p* < 0.05 (significantly different) and *** *p* < 0.01 (highly significantly different). Abbreviations: DG, diacylglycerol; PC, phosphatidylcholine; ChoE, cholesteryl ester; LPC, lysophospholipid; TG, triglyceride; SM, sphingomyelin.

| Metabolites | Predicted healthy  (*n* = 62) | Predicted at-risk of LPL-mediated HTG  (*n* = 44) | *p*-value | *q*-value | VIP |
| --- | --- | --- | --- | --- | --- |
| LPC 16:0 | 42.34 ± 0.8 | 50 ± 1.3 | <0.01*** | <0.01*** | 1.91 |
| LPC 18:0 | 12.59 ± 0.25 | 15.93 ± 0.58 | <0.01*** | <0.01*** | 1.84 |
| TG 52:3 | 55.19 ± 2.86 | 74.16 ± 3.42 | <0.01*** | <0.01*** | 1.68 |
| TG 51:2 | 1.49 ± 0.09 | 1.94 ± 0.1 | <0.01*** | 0.01** | 1.59 |
| ChoE (20:4) | 61.88 ± 1.78 | 77.05 ± 2.4 | <0.01*** | <0.01*** | 1.56 |
| ChoE (18:2) | 122.82 ± 2.51 | 139.24 ± 2.5 | <0.01*** | <0.01*** | 1.55 |
| PC 36:2 | 30.74 ± 0.79 | 35.39 ± 0.97 | <0.01*** | <0.01*** | 1.51 |
| TG 50:3 | 5.44 ± 0.44 | 7.65 ± 0.45 | <0.01*** | <0.01*** | 1.50 |
| TG 52:5 | 2.53 ± 0.17 | 3.58 ± 0.23 | <0.01*** | <0.01*** | 1.49 |
| TG 50:2 | 17.82 ± 1.3 | 23.57 ± 1.39 | <0.01*** | 0.01** | 1.49 |
| TG 52:1 | 3.41 ± 0.31 | 5.61 ± 0.4 | <0.01*** | <0.01*** | 1.47 |
| TG 52:2 | 58.28 ± 3.28 | 74.84 ± 3.53 | <0.01*** | 0.01** | 1.45 |
| PC 38:4 | 10.95 ± 0.35 | 14.12 ± 0.56 | <0.01*** | <0.01*** | 1.45 |
| PC 40:5 | 0.54 ± 0.02 | 0.64 ± 0.02 | <0.01*** | <0.01*** | 1.41 |
| ChoE (16:0) | 6.26 ± 0.14 | 7.23 ± 0.15 | <0.01*** | <0.01*** | 1.41 |
| Valine | 3.29 ± 0.37 | 7.56 ± 2.88 | 0.15 | 0.20 | 1.41 |
| TG 54:4 | 23 ± 1.1 | 29.46 ± 1.39 | <0.01*** | <0.01*** | 1.40 |
| PC 36:4 | 17.59 ± 0.52 | 21.87 ± 0.73 | <0.01*** | <0.01*** | 1.39 |
| PC 40:4 | 0.23 ± 0.01 | 0.29 ± 0.02 | <0.01*** | 0.01** | 1.37 |
| Ornithine | 10.62 ± 0.76 | 27.15 ± 8.09 | 0.05* | 0.09* | 1.37 |
| TG 54:2 | 5.6 ± 0.3 | 7.58 ± 0.39 | <0.01*** | <0.01*** | 1.34 |
| TG 50:4 | 1.02 ± 0.08 | 1.38 ± 0.09 | <0.01*** | 0.02** | 1.34 |
| TG 54:6 | 2.12 ± 0.14 | 2.99 ± 0.2 | <0.01*** | <0.01*** | 1.33 |
| TG 50:1 | 14.15 ± 1.08 | 19.17 ± 1.26 | <0.01*** | 0.01** | 1.32 |
| LPC 16:1 e | 0.29 ± 0.01 | 0.33 ± 0.01 | <0.01*** | 0.01** | 1.32 |
| PC 32:0 | 1.32 ± 0.03 | 1.46 ± 0.03 | <0.01*** | 0.01** | 1.31 |
| PC 38:3 | 4.23 ± 0.14 | 5.17 ± 0.22 | <0.01*** | <0.01*** | 1.30 |
| DG 34:2 | 1.91 ± 0.05 | 2.12 ± 0.04 | <0.01*** | 0.01** | 1.28 |
| Lysine | 2.23 ± 0.31 | 4.93 ± 1.44 | 0.07* | 0.12 | 1.28 |
| SM 42:1 | 17.04 ± 0.45 | 20.19 ± 0.8 | <0.01*** | 0.01** | 1.25 |
| Glutamic acid | 0.09 ± 0.01 | 0.24 ± 0.06 | 0.02** | 0.06* | 1.25 |
| TG 48:2 | 1.72 ± 0.19 | 2.49 ± 0.24 | 0.01** | 0.04** | 1.21 |
| TG 48:1 | 3.43 ± 0.38 | 5.09 ± 0.49 | 0.01** | 0.03** | 1.20 |
| PC 34:0 | 0.34 ± 0.07 | 0.32 ± 0.01 | 0.82 | 0.83 | 1.18 |
| SM 40:1 | 28.54 ± 0.84 | 33.15 ± 1.27 | <0.01*** | 0.01** | 1.17 |
| Tyrosine | 2.13 ± 0.2 | 3.52 ± 0.56 | 0.02** | 0.06* | 1.17 |
| TG 48:3 | 0.46 ± 0.05 | 0.66 ± 0.07 | 0.02** | 0.06* | 1.17 |
| Leucine | 0.25 ± 0.03 | 2.12 ± 1.24 | 0.14 | 0.19 | 1.15 |
| Isoleucine | 0.94 ± 0.11 | 2.1 ± 0.9 | 0.21 | 0.27 | 1.13 |
| DG 34:1 | 3.04 ± 0.09 | 3.4 ± 0.08 | <0.01*** | 0.01** | 1.12 |
| Threonine | 1.55 ± 0.12 | 2.49 ± 0.43 | 0.04** | 0.08* | 1.11 |
| Beta-alanine | 0.54 ± 0.04 | 1.36 ± 0.47 | 0.09* | 0.15 | 1.08 |
| DG 34:3 | 0.38 ± 0.02 | 0.44 ± 0.02 | 0.01** | 0.02** | 1.07 |
| Glycolic acid | 11.16 ± 2.21 | 45.36 ± 15.93 | 0.04** | 0.08* | 1.06 |
| TG 52:6 | 0.33 ± 0.03 | 0.44 ± 0.04 | 0.01** | 0.03** | 1.06 |
| ChoE (18:3) | 15.96 ± 0.64 | 20.36 ± 1.06 | <0.01*** | <0.01*** | 1.05 |
| LPC 20:0 | 0.07 ± 0 | 0.08 ± 0 | 0.01** | 0.03** | 1.03 |
| SM 36:2 | 7.17 ± 0.25 | 8.15 ± 0.28 | 0.01** | 0.03** | 1.03 |
| PC 35:2 | 1.02 ± 0.03 | 1.13 ± 0.03 | 0.02** | 0.06* | 1.02 |
| Tryptophan | 1.41 ± 0.22 | 2.37 ± 0.38 | 0.03** | 0.07* | 1.02 |
| TG 54:3 | 22.92 ± 1.26 | 28.55 ± 1.57 | 0.01** | 0.02** | 1.02 |
| SM 38:1 | 10.82 ± 0.32 | 12.38 ± 0.5 | 0.01** | 0.03** | 1.01 |
| PC 34:1 | 25.06 ± 0.77 | 26.82 ± 0.67 | 0.08* | 0.14 | 1.01 |
| ChoE (18:1) | 26.27 ± 0.64 | 28.56 ± 0.58 | 0.01** | 0.03** | 1.01 |
| LPC 16:0 e | 0.31 ± 0.01 | 0.34 ± 0.01 | 0.02** | 0.05* | 0.99 |
| PC 38:2 | 0.47 ± 0.01 | 0.51 ± 0.01 | 0.03** | 0.06* | 0.98 |
| ChoE (18:0) | 0.58 ± 0.02 | 0.71 ± 0.03 | <0.01*** | <0.01*** | 0.96 |
| SM 35:1 | 2.38 ± 0.08 | 2.7 ± 0.1 | 0.01** | 0.04** | 0.95 |
| Hydroxyproline | 0.49 ± 0.06 | 2.12 ± 0.94 | 0.09* | 0.15 | 0.95 |
| SM 34:1 | 94.82 ± 2.5 | 103.87 ± 2.79 | 0.02** | 0.05* | 0.95 |
| Aspartic acid | 0.62 ± 0.13 | 1.31 ± 0.35 | 0.07* | 0.12 | 0.95 |
| SM 36:1 | 14.43 ± 0.46 | 16.29 ± 0.57 | 0.01** | 0.04** | 0.94 |
| SM 43:1 | 1.01 ± 0.03 | 1.13 ± 0.04 | 0.01** | 0.04** | 0.93 |
| Asparagine | 0.15 ± 0.02 | 0.34 ± 0.09 | 0.05* | 0.09* | 0.92 |
| LPC 18:2 | 18.45 ± 0.68 | 20.33 ± 0.98 | 0.12 | 0.17 | 0.92 |
| SM 41:1 | 11.7 ± 0.33 | 12.95 ± 0.45 | 0.03** | 0.07* | 0.91 |
| LPC 18:1 | 10.3 ± 0.33 | 10.87 ± 0.44 | 0.30 | 0.35 | 0.91 |
| LPC 18:0 e | 0.08 ± 0 | 0.09 ± 0 | 0.03** | 0.07* | 0.88 |
| ChoE (22:6) | 15.17 ± 0.57 | 17.77 ± 0.78 | 0.01** | 0.03** | 0.85 |
| SM 40:2 | 12.51 ± 0.39 | 13.96 ± 0.54 | 0.03** | 0.07* | 0.85 |
| DG 36:4 | 1.66 ± 0.07 | 1.89 ± 0.07 | 0.03** | 0.06* | 0.84 |
| Cholesterol | 0.36 ± 0.05 | 0.88 ± 0.31 | 0.11 | 0.16 | 0.84 |
| ChoE (22:5) | 0.61 ± 0.02 | 0.68 ± 0.03 | 0.05* | 0.09* | 0.84 |
| Glycine | 8.95 ± 0.93 | 29.08 ± 11.8 | 0.10 | 0.15 | 0.83 |
| ChoE (16:1) | 3.14 ± 0.17 | 3.43 ± 0.23 | 0.31 | 0.36 | 0.83 |
| Phenylalanine | 1.18 ± 0.08 | 1.68 ± 0.26 | 0.06* | 0.11 | 0.83 |
| 3-hydroxybutiric acid | 1.48 ± 0.11 | 1.17 ± 0.09 | 0.03** | 0.07* | 0.82 |
| PC 32:2 | 0.37 ± 0.02 | 0.41 ± 0.02 | 0.14 | 0.19 | 0.81 |
| SM 42:2 | 44.05 ± 1.4 | 47.35 ± 1.37 | 0.10 | 0.15 | 0.81 |
| TG 46:1 | 0.45 ± 0.07 | 0.66 ± 0.12 | 0.12 | 0.17 | 0.81 |
| Glutamine | 1.51 ± 0.1 | 2.23 ± 0.31 | 0.03** | 0.07* | 0.80 |
| TG 46:2 | 0.23 ± 0.03 | 0.33 ± 0.05 | 0.09* | 0.15 | 0.80 |
| PC 32:1 | 1.49 ± 0.09 | 1.62 ± 0.11 | 0.36 | 0.40 | 0.80 |
| TG 48:0 | 1.12 ± 0.1 | 1.41 ± 0.15 | 0.11 | 0.16 | 0.79 |
| SM 42:3 | 22.22 ± 0.62 | 23.64 ± 0.66 | 0.12 | 0.17 | 0.75 |
| ChoE (17:1) | 0.17 ± 0.01 | 0.18 ± 0.01 | 0.18 | 0.24 | 0.75 |
| SM 34:2 | 10.78 ± 0.3 | 11.65 ± 0.34 | 0.06* | 0.11 | 0.75 |
| PC 30:0 | 0.2 ± 0.01 | 0.24 ± 0.02 | 0.09* | 0.14 | 0.75 |
| DG 36:2 | 6.04 ± 0.18 | 6.75 ± 0.22 | 0.01** | 0.04** | 0.72 |
| ChoE (22:4) | 0.15 ± 0.01 | 0.2 ± 0.02 | 0.02** | 0.06* | 0.71 |
| LPC 15:0 | 0.3 ± 0.01 | 0.32 ± 0.01 | 0.22 | 0.28 | 0.69 |
| Proline | 0.3 ± 0.03 | 0.59 ± 0.13 | 0.04** | 0.08* | 0.65 |
| SM 33:1 | 2.97 ± 0.09 | 3.26 ± 0.12 | 0.05* | 0.10 | 0.64 |
| Alanine | 1.48 ± 0.15 | 3.43 ± 1.36 | 0.16 | 0.22 | 0.63 |
| ChoE (20:2) | 0.35 ± 0.02 | 0.41 ± 0.02 | 0.05* | 0.10 | 0.63 |
| TG 54:7 | 0.94 ± 0.08 | 1.2 ± 0.12 | 0.08* | 0.14 | 0.62 |
| SM 32:1 | 5.93 ± 0.2 | 6.54 ± 0.27 | 0.07* | 0.12 | 0.61 |
| Serine | 0.2 ± 0.02 | 0.32 ± 0.06 | 0.05* | 0.09* | 0.61 |
| Histidine | 0.08 ± 0.02 | 0.3 ± 0.19 | 0.26 | 0.32 | 0.58 |
| Ribose | 0.24 ± 0.01 | 0.22 ± 0.02 | 0.17 | 0.23 | 0.57 |
| TG 46:0 | 0.59 ± 0.05 | 0.63 ± 0.08 | 0.67 | 0.69 | 0.57 |
| PC 31:0 | 0.07 ± 0 | 0.07 ± 0 | 0.19 | 0.25 | 0.56 |
| SM 39:1 | 4.15 ± 0.14 | 4.56 ± 0.21 | 0.11 | 0.16 | 0.55 |
| Methionine | 0.17 ± 0.03 | 0.31 ± 0.11 | 0.23 | 0.28 | 0.55 |
| Malic acid | 0.34 ± 0.11 | 0.49 ± 0.15 | 0.42 | 0.46 | 0.55 |
| Lactic acid | 3.9 ± 0.09 | 4.07 ± 0.12 | 0.27 | 0.32 | 0.54 |
| Glycerol | 9.07 ± 5.88 | 18.93 ± 9.56 | 0.38 | 0.43 | 0.51 |
| Pyruvic acid | 16.21 ± 0.84 | 16.18 ± 0.74 | 0.98 | 0.98 | 0.46 |
| PC 33:1 | 0.24 ± 0.01 | 0.25 ± 0.01 | 0.60 | 0.63 | 0.44 |
| PC 33:0 | 0.05 ± 0 | 0.05 ± 0 | 0.26 | 0.32 | 0.40 |
| SM 41:2 | 6.42 ± 0.19 | 6.76 ± 0.25 | 0.29 | 0.35 | 0.39 |
| Glyceric acid | 0.57 ± 0.02 | 0.56 ± 0.02 | 0.58 | 0.62 | 0.36 |
| Glucose | 0.29 ± 0.01 | 0.32 ± 0.02 | 0.11 | 0.16 | 0.33 |
| TG 50:0 | 0.38 ± 0.04 | 0.51 ± 0.06 | 0.07* | 0.12 | 0.27 |
| Alpha-tocopherol | 0.36 ± 0.03 | 0.42 ± 0.05 | 0.36 | 0.40 | 0.25 |
| Alpha-ketoglutarate | 0.5 ± 0.02 | 0.53 ± 0.03 | 0.48 | 0.51 | 0.24 |
| Threonic acid | 1.66 ± 0.06 | 1.55 ± 0.1 | 0.38 | 0.43 | 0.21 |
| Oleic acid | 3.5 ± 0.15 | 3.9 ± 0.37 | 0.32 | 0.37 | 0.21 |
| Fructose | 0.35 ± 0.04 | 0.32 ± 0.02 | 0.42 | 0.46 | 0.19 |
| Aconitic acid | 0.02 ± 0 | 0.02 ± 0 | 0.40 | 0.44 | 0.18 |
| Urea | 1.2 ± 0.04 | 1.23 ± 0.04 | 0.64 | 0.66 | 0.18 |
| Citric acid | 5.84 ± 0.19 | 5.95 ± 0.25 | 0.72 | 0.74 | 0.16 |
| 2-hydroxyglutaric | 0.97 ± 0.04 | 0.89 ± 0.05 | 0.23 | 0.28 | 0.10 |
| Fumaric acid | 0.48 ± 0.02 | 0.46 ± 0.02 | 0.47 | 0.51 | 0.08 |
| ChoE (17:0) | 0.07 ± 0.01 | 0.07 ± 0.02 | 0.69 | 0.70 | 0.04 |
| Succinic acid | 0.63 ± 0.03 | 0.58 ± 0.03 | 0.21 | 0.27 | 0.01 |

**Supplementary Figure 5. PCA summary plot of the plasma metabolome of healthy human population (according to TG guidelines) classified as healthy and at-risk of LPL-mediated HTG (predictive model).** (a) Explained variance. The scree plot suggests that 5 components may be sufficient to capture most of the variance. (b) Observation diagnostics. This plot shows the distances within and orthogonal to the projection plane; the names of the samples with a high value for at least one of the distances are given. (c) Score plot (PCA). The total variance explained is 28%: PC1 explains the 15% and the PC2 explains the 13%. (d) The variables with the most extreme values (positive and negative) for each loading are coloured black and labelled. Legend: red, healthy; blue, at-risk of LPL-mediated HTG.

**Supplementary Figure 6. OPLS-DA of the plasma metabolome of the human cohort (according to TG guidelines) classified as healthy and at-risk of LPL-mediated HTG (predictive model).** (a) Overview of the model. The graph here suggests that 2 components may be sufficient to capture most of the inertia (b) Diagnostic significance. The R2Y and Q2Y of the model are compared with the corresponding values obtained after random permutation of the y-response. (c) Observation diagnostics. Indicates possible outliers. (d) X-score plot (OPLS-DA). The graph shows the number of components, the cumulative explained variance in X (R2X), in Y (R2Y), and the cumulative prediction power (Q2Y), the root mean square error of estimation (RMSEE), the predicted variation in Y (pre), and the orthogonal variance in Y (ort). Legend: red, healthy; blue, at-risk of LPL-mediated HTG.
